# Loss-of-function variants in the schizophrenia risk gene *SETD1A* alter neuronal network activity in human neurons through the cAMP/PKA pathway

**DOI:** 10.1016/j.celrep.2022.110790

**Published:** 2022-05-03

**Authors:** Shan Wang, Jon-Ruben van Rhijn, Ibrahim Akkouh, Naoki Kogo, Nadine Maas, Anna Bleeck, Irene Santisteban Ortiz, Elly Lewerissa, Ka Man Wu, Chantal Schoenmaker, Srdjan Djurovic, Hans van Bokhoven, Tjitske Kleefstra, Nael Nadif Kasri, Dirk Schubert

**Affiliations:** 1 Department of Cognitive Neurosciences, Radboudumc, Donders Institute for Brain Cognition and Behaviour, 6525 HR Nijmegen, the Netherlands; 2 Department of Medical Genetics, Oslo University Hospital, 0424 Oslo, Norway; 3 NORMENT, Institute of Clinical Medicine, University of Oslo, 0372 Oslo, Norway; 4 Department of Human Genetics, Radboudumc, Donders Institute for Brain Cognition and Behaviour, 6500 HB Nijmegen, the Netherlands; 5 Department of Biophysics, Donders Institute for Brain Cognition and Behaviour, 6525 AJ Nijmegen, the Netherlands; 6 NORMENT, Department of Clinical Science, University of Bergen, 5021 Bergen, Norway

## Abstract

Heterozygous loss-of-function (LoF) mutations in *SETD1A*, which encodes a subunit of histone H3 lysine 4 methyltransferase, cause a neurodevelopmental syndrome and increase the risk for schizophrenia. Using CRISPR-Cas9, we generate excitatory/inhibitory neuronal networks from human induced pluripotent stem cells with a *SETD1A* heterozygous LoF mutation (*SETD1A*^+/−^). Our data show that *SETD1A* haploinsufficiency results in morphologically increased dendritic complexity and functionally increased bursting activity. This network phenotype is primarily driven by *SETD1A* haploinsufficiency in glutamatergic neurons. In accordance with the functional changes, transcriptomic profiling reveals perturbations in gene sets associated with glutamatergic synaptic function. At the molecular level, we identify specific changes in the cyclic AMP (cAMP)/Protein Kinase A pathway pointing toward a hyperactive cAMP pathway in *SETD1A*^+/−^ neurons. Finally, by pharmacologically targeting the cAMP pathway, we are able to rescue the network deficits in *SETD1A*^+/−^ cultures. Our results demonstrate a link between SETD1A and the cAMP-dependent pathway in human neurons.

## Graphical abstract

**Figure:**
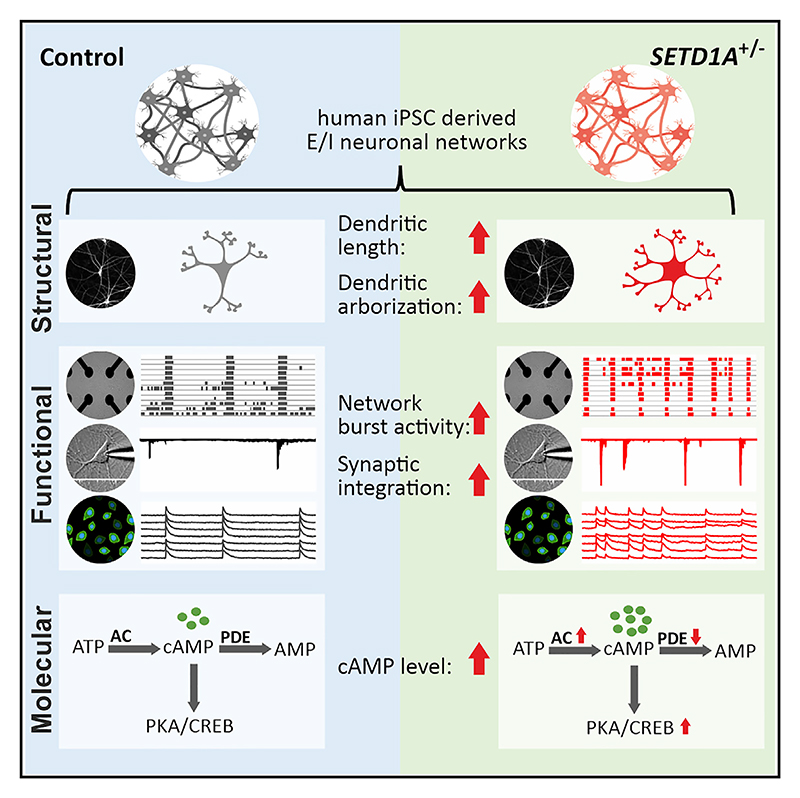

## Introduction

Schizophrenia (SCZ) is a complex and heterogeneous syndrome with poorly defined neurobiology. It is a highly heritable disease (~80% heritability) with a substantial genetic component (Hilker et al., 2018; Legge et al., 2021; Skene et al., 2018). In the past decade, considerable progress has been made to better understand the genetic burden related to SCZ. Genetic loci related to SCZ can be common variants, which typically have small effects, or rare variants, which can result in a large effect on individual risk (Legge et al., 2021). One of the genes with such rare large-effect size variants is *SETD1A*, encoding SET domain-containing protein 1A. Studies have demonstrated that loss-of-function (LoF) and missense mutations in *SETD1A* are associated with SCZ (Singh et al., 2016, 2022; Takata et al., 2014) but also found in individuals with disrupted speech development (Eising et al., 2019) and early-onset epilepsy (Yu et al., 2019). We have presented a series of *de novo SETD1A* heterozygous LoF mutations and defined a neurodevelopmental syndrome based on a cohort of 15 individuals (age ranging from 34 months to 23 years) (Kummeling et al., 2020). The core characteristics of these individuals include global developmental delay (such as speech delay or motor delay) and/or intellectual disability, facial dysmorphisms, as well as behavior and psychiatric abnormalities, including psychotic episodes (2 of 15 individuals). In addition, abnormalities in brain structure and visual and hearing impairments have also been reported in some of these individuals (Kummeling et al., 2020). *SETD1A* mutations thus appear to cause biological vulnerability to a broad neurodevelopmental phenotypic spectrum.

*SETD1A* encodes a subunit of the human Set/COMPASS complex (complex of proteins associating with Set1), which methylates histone H3 at position lysine 4 (H3K4me1, H3K 4me2, and H3K4me3) and participates in regulation of gene expression. Mouse models with heterozygous LoF mutation of *Setd1a* (*Setd1a*^+/−^) recapitulate SCZ-related behavioral abnormalities, such as deficits in working memory and social interaction (Mukai et al., 2019; Nagahama et al., 2020). At the cellular level, *Setd1a*^+/−^ mice display reduced axon branches and dendritic spines, increased neuronal excitability (Mukai et al., 2019), as well as impaired excitatory synaptic neurotransmission (Nagahama et al., 2020). *Setd1a*^+/−^ mice show an altered transcriptomic profile in the medial prefrontal cortex (mPFC), a highly relevant region for SCZ (Mukai et al., 2019). In other brain regions, such as the primary visual cortex (V1), *Setd1a*^+/−^ mice exhibit aberrant ensemble activity and gamma oscillations (Hamm et al., 2020). All of these studies suggest that *SETD1A* haploinsufficiency results in neuronal circuit dysfunction. However, the exact cellular and molecular mechanisms of how *SETD1A* mutations lead to disrupted neuronal connectivity, causing such severe mental symptoms, especially in a human context, remain poorly understood.

To investigate the role of SETD1A in neuronal network development and synaptic organization, we generated an isogenic human induced pluripotent stem cell (hiPSC) line with *SETD1A* haploinsufficiency through CRISPR-Cas9. Subsequently, hiPSCs were differentiated into homogeneous populations of glutamatergic and Gamma aminobutyric acid (GABA)-ergic neurons (Mossink et al., 2021). In *in vitro* cultures containing defined compositions of glutamatergic and GABAergic neurons, we comprehensively analyzed molecular, structural, and functional neuronal properties from single cells and neuronal networks during development. The results presented here demonstrate that *SETD1A* haploinsufficiency leads to key morphological, electrophysiological, and transcriptional alterations. At the molecular level, we show that the *SETD1A*^+/−^ network phenotype is mediated by an upregulated cyclic adenosine monophosphate (cAMP)/Protein Kinase A (PKA) pathway. This was confirmed by showing that pharmacological inhibition of cAMP/PKA rescues the *SETD1A*^+/−^ network phenotype. Therefore, our results reveal cAMP/PKA as a potential down-stream pathway affected by *SETD1A* mutation, opening therapeutic opportunities for individuals carrying the *SETD1A* variant.

## Results

### *SETD1A*^+/−^ neuronal networks exhibit dysregulated functional organization

**Figure 1:**
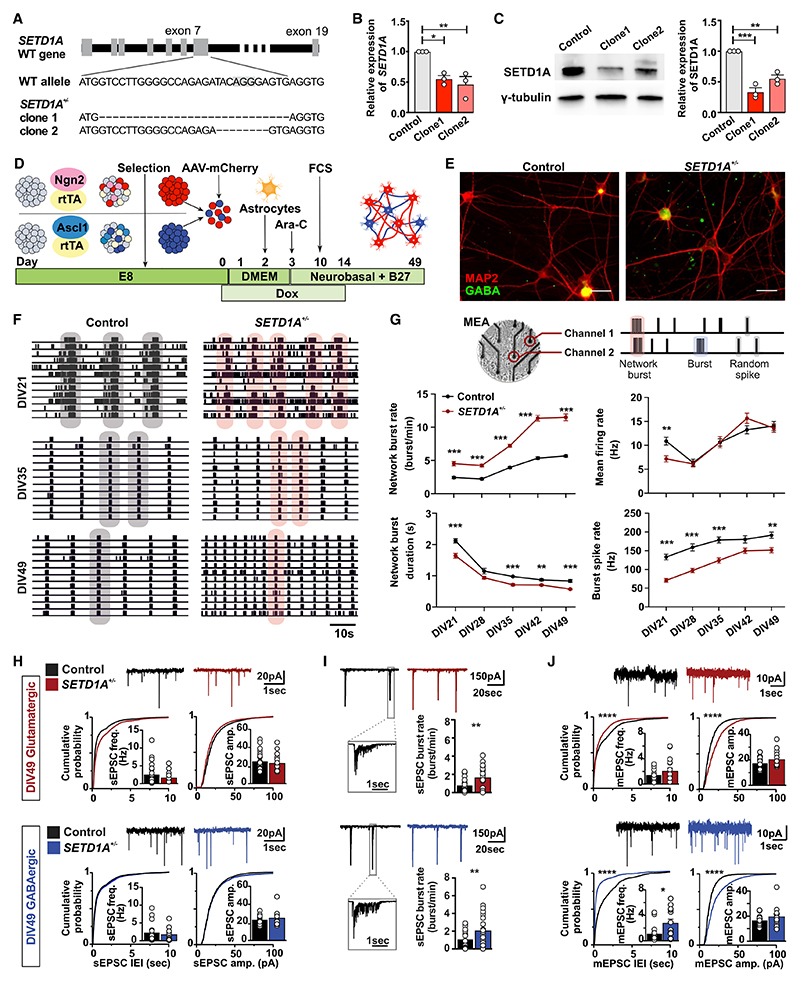
*SETD1A*^+/−^ neuronal networks exhibit dysregulated functional organization (A) Generation of *SETD1A* isogenic lines (*SETD1A*^*+/*−^) using CRISPR-Cas9. The schematic shows the position of the single-guide RNA (sgRNA) sequence and indels generated in *SETD1A*^+/−^ clone 1 and *SETD1A*^+/−^ clone 2. (B and C) qPCR and western blot showing reduced mRNA (B) and protein levels of SETD1A (C). n = 3 for each group. (D) Schematic representation of the neuronal differentiation workflow. (E) Immunofluorescence staining of GABA (green) and MAP2 (red) at DIV49. Scale bar, 30 μm. (F and G) Analyses of neuronal activity using MEA recordings. (F) Representative raster plots (1 min) of electrophysiological activity exhibited by control and *SETD1A*^*+/*−^ neuronal networks at different time points during development. Gray and pink shadows show examples of network burst events. (G) Schematic overview of an electrophysiological recording from neurons cultured on MEA (top panel) and quantification of network parameters as indicated (bottom panel). Sample size: control n = 40 MEA wells, *SETD1A*^*+/*−^ n = 63 (clone 1 = 15, clone 2 = 48) MEA wells from 5 independent batches. (H and I) Representative whole-cell voltage-clamp recordings and quantitative analyses of (H) spontaneous excitatory postsynaptic currents (sEPSCs) and (I) correlated synaptic inputs (sEPSC bursts) in glutamatergic and GABAergic neurons in control and *SETD1A*^*+/*−^ E/I cultures at DIV49. sEPSC frequency and amplitude: glutamatergic neurons: control n = 21 cells; *SETD1A*^*+/*−^ n = 19 cells (clone 1 = 13, clone 2 = 6); GABAergic neurons: control n = 18 cells, *SETD1A*^*+/*−^ n = 16 cells (clone 1 = 11, clone 2 = 5). sEPSC burst: glutamatergic neurons: control n = 24 cells, *SETD1A*^*+/*−^ n = 24 cells (clone 1 = 14, clone 2 = 10); GABAergic neurons: control n = 22 cells, *SETD1A*^*+/*−^ n = 22 cells (clone 1 = 11, clone 2 = 11). (J) Representative whole-cell voltage-clamp recordings and quantitative analyses of mEPSC activity in glutamatergic and GABAergic neurons. Glutamatergic neurons: control n = 17 cells, *SETD1A*^*+/*−^ n = 16 cells from clone 1; GABAergic neurons: control n = 16 cells, *SETD1A*^*+/*−^ n = 13 cells from clone 1. Data represent mean ± SEM. *p < 0.05, **p < 0.01, ***p < 0.001, two-way ANOVA with post hoc Bonferroni correction (G), Student’s t test with Bonferroni correction for multiple testing (H–J), or Kolmogorov-Smirnov test (H) for comparing control and *SETD1A*^*+/*−^ cultures.

We used CRISPR-Cas9 to generate an isogenic hiPSC line with a heterozygous LoF mutation of *SETD1A* by targeting exon 7 of *SETD1A* in a healthy hiPSC line (Miyaoka et al., 2014; Figure 1A). We introduced a frameshift mutation in exon 7 leading to LoF of the protein, mimicking a mutation reported in an individual diagnosed with SCZ (Takata et al., 2014). Two clones with insertions or deletions (indels) in *SETD1A* were selected for further characterization, which carried 28-bp (clone 1) and 8-bp (clone 2) deletions on one allele, respectively (Figures 1A and S1A), both predicting a premature stop codon. SETD1A mRNA and protein levels were approximately halved in both *SETD1A*^+/−^ hiPSC clones, indicating that both variants represent LoF alleles (Figures 1B, 1C, and S1B). All selected clones showed positive expression of pluripotency markers (OCT4, TRA-1-81, NANOG, and SSEA4), and karyotyping and off-target analysis were performed to confirm genetic integrity (Figures S1C–S1E).

Disrupted neuronal connectivity has been reported in individuals with SCZ and animal models of SCZ (Owen et al., 2016). We evaluated whether LoF of the *SETD1A* gene results in neuronal network impairment *in vitro*. We generated composite networks of glutamatergic (~75%) and GABAergic (~25%) neurons (excitatory/inhibitory [E/I] cultures) comprised of control or *SETD1A*^+/−^ hiPSCs by forced expression of the transcription factor *Ngn2* or *Ascl1*, respectively, as described previously (Figure 1D; Mossink et al., 2021). We did not detect any significant differences in the percentage of glutamatergic or GABAergic neurons between control and *SETD1A*^+/−^ networks at day *in vitro* (DIV) 49 (Figures 1E and S2A). After acute treatment with 100 μM picrotoxin (PTX) at DIV49 on a microelectrode array (MEA), mean firing rate and network burst duration increased for control and *SETD1A*^+/−^ networks (Figure S2B), indicating that, at DIV49, GABAergic neurons exhibit robust inhibitory control (Mossink et al., 2021). In addition, we found that *SETD1A*^+/−^ neurons exhibited reduced expression of H3K4me3 (Figure S1F), which is in line with the function of SETD1A as a histone methyltransferase.

To assess whether neuronal network activity differs between control and *SETD1A*^+/−^ E/I cultures during development, we used MEA recordings (Frega et al., 2019; Figure 1G). We recorded neuronal network activity once a week from DIV21 to DIV49. After 3 weeks of differentiation, control and *SETD1A*^+/−^ networks showed network burst activity (Figure 1F), which indicated that the neurons were functionally connected and integrated into a network. Network burst activity increased during development for both groups and plateaued around DIV42. Strikingly, *SETD1A*^+/−^ networks showed significantly increased network burst activity compared with the control from DIV21 to DIV49, accompanied by a shorter network inter-burst interval, a decrease in network burst duration, as well as a lower spike rate within a burst (Figures 1G and S2C). Interestingly, the global activity (i.e., the mean firing rate; Figure 1G) was similar in control and *SETD1A*^+/−^ networks, which implies that there was functional re-organization of network connectivity in *SETD1A*^+/−^ E/I cultures rather than general hyperactivity. Calcium imaging confirmed the increased synchronized activity in *SETD1A*^+/−^ networks (Figures S2D–S2G; Videos S1 and S2). We corroborated the network phenotype in a second CRISPR *SETD1A*^+/−^ line derived from a healthy female donor, excluding any confounding bias resulting from genetic background or gender effect (Figures S2H–S2K).

We next evaluated whether this functional network re-organization in *SETD1A*^*+/*−^ E/I cultures is related to changes in intrinsic properties and/or synaptic inputs using single-cell patch clamping. Intrinsic properties were similar in control and *SETD1A*^*+/*−^ neurons at DIV21 and DIV49 (Figures S3A and S3B; Table S1) in glutamatergic and GABAergic neurons. We also measured general synaptic integration by recording spontaneous excitatory postsynaptic currents (sEPSCs). Neither sEPSC frequency nor amplitude was different between control and *SETD1A*^*+/*−^ cultures at DIV21 or DIV49 (Figure 1H; Table S1). However, we did observe that the frequency of temporally correlated bursts of synaptic inputs (sEPSC bursts) onto the postsynaptic neuron was increased significantly at DIV49 in *SETD1A*^+/−^ E/I cultures on glutamatergic and GABAergic neurons (Figure 1I). This is in line with the increased network burst rate at the population level shown in the MEA recordings and confirms that reduced SETD1A expression results in increased network activity. Because analysis of sEPSCs reflects network activity rather than yielding quantitative information regarding synaptic connectivity, we next measured miniature EPSCs (mEPSCs), which are action potential independent and can be indicative of reorganization of synaptic inputs, altered synapse numbers, and changes in release probabilities (mEPSC frequency) and receptor abundance (mEPSC amplitude). mEPSC frequency and amplitude were increased significantly in glutamatergic and GABAergic *SETD1A*^*+/*−^ neurons at DIV49 (Figure 1J). This suggests that networks comprised of *SETD1A*^*+/*−^ neurons not only exhibit increased network activity but also elevated synaptic connectivity, and this could be a major contributor to the network phenotype we observed at the population level.

### *SETD1A*^+/−^ neurons show aberrant somatodendritic morphology

**Figure 2:**
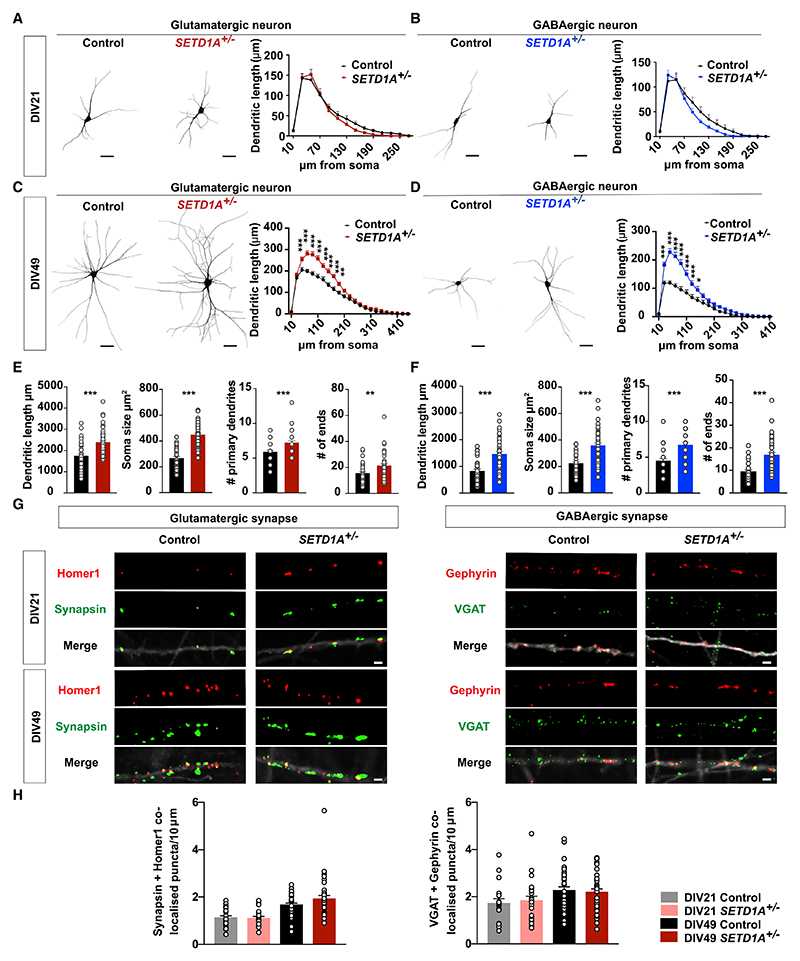
*SETD1A*^+/−^ neurons show aberrant somatodendritic morphology (A and B) Representative somatodendritic reconstructions of glutamatergic (A) and GABAergic (B) neurons and Sholl analysis in control and *SETD1A*^+/−^ neurons at DIV21 (glutamatergic neurons: n = 20 for control, n = 20 for *SETD1A*^+/−^ from clone 1; GABAergic neurons: n = 18 for control, n = 21 for *SETD1A*^+/−^ from clone 1). Dendritic length represents the length of dendrites that occur at fixed distances from the soma in concentric circles. Scale bars, 40 μm. (C and D) Representative somatodendritic reconstructions of glutamatergic (C) or GABAergic (D) neurons and Sholl analysis in control and *SETD1A*^+/−^ networks at DIV49 (glutamatergic neurons: n = 38 for control, n = 47 for *SETD1A*^+/−^ [clone 1 = 23, clone 2 = 24]; GABAergic neurons: n = 33 for control, n = 45 for *SETD1A*^+/−^ [clone 1 = 22, clone 2 = 23]). Scale bars, 40 μm. (E and F) Main morphological parameters in reconstruction for glutamatergic neurons (E) and GABAergic neurons (F) at DIV49. (G) Representative images of immunocytochemistry stained for glutamatergic synapse (synapsin as a presynaptic marker and Homer1 as a postsynaptic marker) and GABAergic synapse (VGAT as a pre-synaptic marker and Gephyrin as a post-synaptic marker). Scale bar, 2 μm. (H) Quantification of the density of co-localized Synapsin/Homer1 and VGAT/Gephyrin puncta (number per 10 μm). Synapsin/Homer1: n = 25 for control, n = 19 for *SETD1A*^+/−^ at DIV21 from clone 1; n = 34 for control, n = 36 for *SETD1A*^+/−^ at DIV49 (clone 1 = 26, clone 2 = 10); VGAT/Gephyrin: n = 18 for control, n = 23 for *SETD1A*^+/−^ at DIV21 from clone 1; n = 34 for control, n = 36 for *SETD1A*^+/−^ at DIV49 (clone 1 = 26, clone 2 = 10). Data represent mean ± SEM. *p < 0.05, **p < 0.01, ***p < 0.001, two-way ANOVA with post hoc Bonferroni correction (A–D), unpaired Student’s t test (E and F), or one-way ANOVA with post hoc Bonferroni correction (H).

Based on the MEA results, we next sought to investigate whether *SETD1A*^+/−^ neurons exhibit changes in dendritic morphology and/or synapse formation. At DIV21, control and *SETD1A*^+/−^ E/I cultures did not differ from each other in any of the morphological parameters for glutamatergic and GABAergic neurons (Figures 2A and 2B). However, at DIV49, *SETD1A*^+/−^ neurons displayed a significantly larger soma size, accompanied by longer dendritic length, more dendritic branches, and a larger covered area for glutamatergic and GABAergic neurons (Figures 2C–2F). Finally, we performed a Sholl analysis and confirmed that there were no significant differences in the distribution of dendritic length at DIV21 for both neuronal types (Figures 2A, 2B, S4A, and S4B), whereas, at DIV49, the dendritic length at multiple distances from the soma was significantly longer in glutamatergic and GABAergic *SETD1A*^+/−^ neurons (Figures 2C, 2D, S4C, and S4D). These results indicate that *SETD1A* haploinsufficiency leads to a more complex somatodendritic morphology, but this only becomes significant during later developmental stages.

To measure synapse formation, we immunostained neurons for pre- and postsynaptic markers (Synapsin and Homer1 for glutamatergic synapses and the vesicular GABA transporter (VGAT) and Gephyrin for GABAergic synapses). We found no differences in the density of Synapsin/Homer1 or VGAT/Gephyrin co-localized puncta between genotypes at DIV21 and DIV49 on excitatory and inhibitory neurons (Figures 2G, 2H, S4E, and S4F). These data, combined with the observation that all *SETD1A*^*+/*−^ neurons show a longer dendritic length at DIV49, imply that the total amount of synapses per neuron is higher in *SETD1A*^+/−^ neurons, which is reflected by the increase in mEPSC frequency at DIV49 (Figure 1J).

### *SETD1A* haploinsufficiency leads to an altered transcriptomic profile

**Figure 3:**
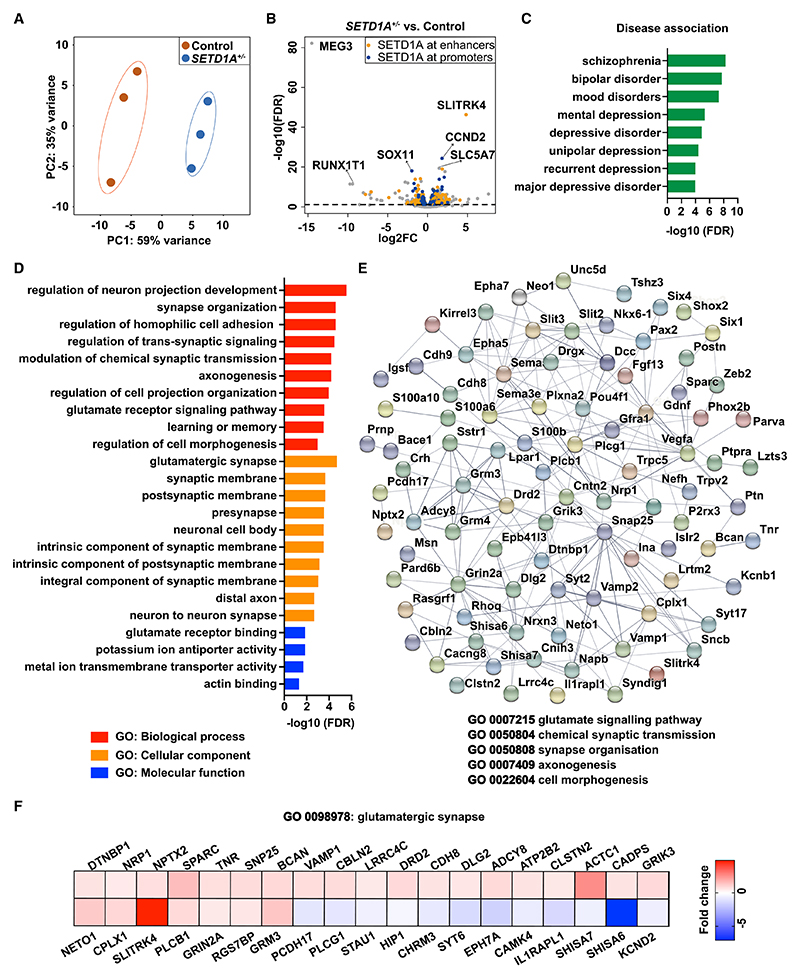
*SETD1A* haploinsufficiency leads to an altered transcriptomics profile (A) PCA showing tight clustering of 3 replicates for each genotype. *SETD1A*^+/−^ was from clone 1. (B) Volcano plots showing differentially expressed genes (DEGs) between *SETD1A*^*+/*−^ and control E/I cultures. Relative to the control, significantly up- or down-regulated genes are shown above the black dashed line. Among these 919 DEGs, 556 (61%) genes are predicted to be SETD1A target genes (labeled in orange and blue) by comparing our DEGs with the published ChIP-seq database of SETD1A (Mukai et al., 2019). The top 3 upregulated and downregulated DEGs are labeled with the gene name. (C) Disease terms of the DisGeNET database associated with DEGs. (D) Gene Ontology (GO) term analysis of DEGs. (E) Diagram of the dysregulated protein network, showing the interactions among several synaptic functions using STRING. (F) Heatmap showing the fold change of DEGs compared with the control in gene sets related to glutamatergic synapses.

We showed that *SETD1A*^+/−^ neurons exhibited reduced expression of H3K4me3, which is expected to result in alterations in the transcriptomic profile and, consequently, altered neuronal network phenotypes. To identify the molecular perturbations that underlie the network phenotypes caused by *SETD1A* haploinsufficiency, we conducted RNA sequencing (RNA-seq) at DIV49. Principal-component analysis (PCA) of transcriptional profiles exhibited clear clustering of biological replicates per genotype (Figure 3A). We quantified the differentially expressed genes (DEGs) and found that 380 genes were downregulated and 539 genes were upregulated in *SETD1A*^+/−^ E/I cultures compared with control cultures (Figure 3B). To further explore whether the DEGs are SETD1A target genes, we compared our DEGs with the chromatin immunoprecipitation sequencing (ChIP-seq) database of SETD1A from a recently published study (Mukai et al., 2019). The results show that SETD1A binds to enhancer or promoter regions of 556 DEGs (61% of total DEGs) (Figure 3B; Data S2). Interestingly, when we performed a disease enrichment analysis using the DisGeNET database, which consists of more than 10,000 disorders (Piñero et al., 2020), SCZ was identified as the top hit (Figure 3C; Table S2). This indicates that *SETD1A* haploinsufficiency in our model captures some key genetic alterations linked to SCZ. In addition, we conducted Gene Ontology (GO) analysis to examine which biological functions are over-represented in the DEGs. Significant enrichment in GO terms relevant to “synaptic function,” “morphogenesis,” “ion channels,” and “learning and memory” were identified (Figure 3D; Table S2). Proteins encoded by these genes interact with each other, as indicated in the protein-protein interaction analysis generated through Search Tool for the Retrieval of Interacting Genes (STRING) (Szklarczyk et al., 2021) (Figure 3E). Interestingly, we noticed that many GO terms and DEGs relevant to glutamatergic neurons were identified, such as “glutamate receptor binding,” “glutamate receptor signaling pathway,” and “glutamatergic synapse” (Figures 3D–3F). This is consistent with the data from *Setd1a*^*+/*−^ mouse models, where it has been shown that SETD1A target genes are highly expressed in pyramidal neurons (Mukai et al., 2019). This also suggests that the altered network phenotype caused by *SETD1A* haploinsufficiency might be driven predominantly by perturbations in glutamatergic neurons.

To explore the cross-species validity of the SETD1A target genes, we compared our DEGs with the published transcriptome of *Setd1a*^*+/*−^ mouse model from Mukai et al. (2019) and found, in total, 11 overlapping genes (Table S2). One of these genes is *SLITRK4*, the gene showing the strongest upregulation in *SETD1A*^+/−^ cultures (Figure 3B), which is involved in neurite outgrowth (Yim et al., 2013). We also found a significant association between our DEGs and 78 SFARI genes (Fisher’s exact test, p = 1.1e^–5^), an autism-related genes database (Table S2). This is in line with the suggested genetic overlap between SCZ and autism (Carroll and Owen, 2009). These results indicate that transcription is profoundly disturbed in *SETD1A*^+/−^ neurons and that DEGs are enriched in specific gene sets related to SCZ and synaptic function, especially the ones relevant for glutamatergic synapse function.

### *SETD1A*^+/−^ glutamatergic neuronal cultures recapitulate the *SETD1A*^+/−^ E/I network phenotype

**Figure 4:**
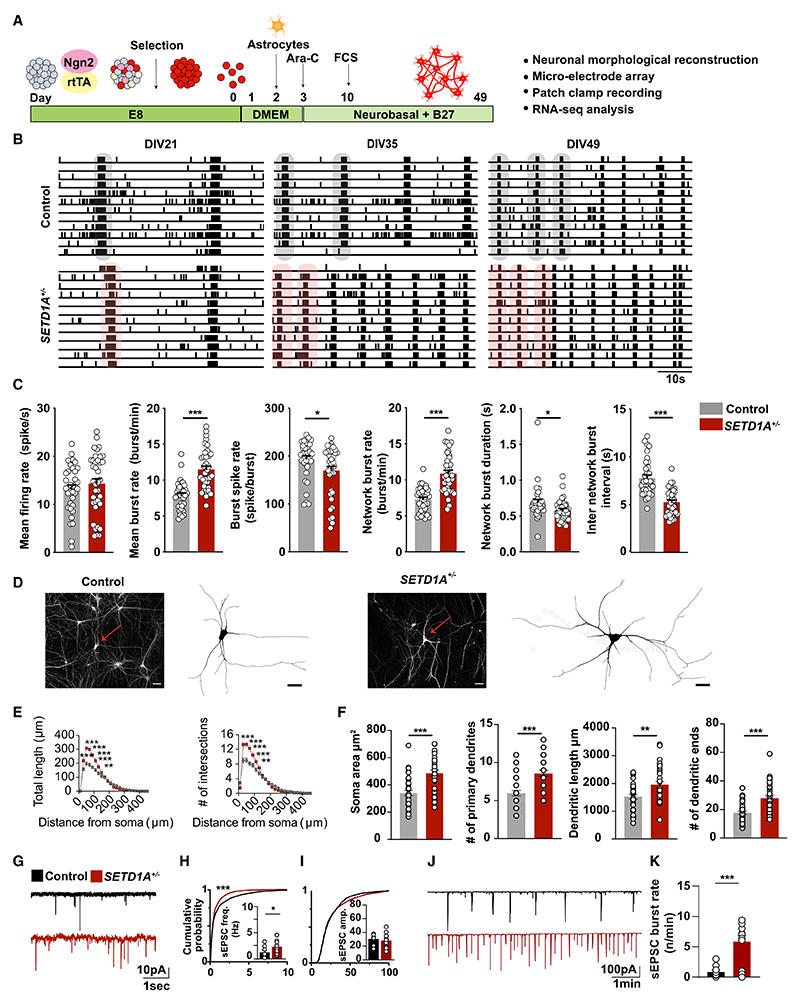
*SETD1A*^+/−^ glutamatergic neuronal cultures recapitulate the *SETD1A*^+/−^ E/I network phenotype (A) Schematic of the neuronal differentiation workflow. (B) Representative raster plots (1 min) of electrophysiological activity measured by MEA from control and *SETD1A*^+/−^ glutamatergic neuronal networks at different time points during development. (C) Quantification of network parameters as indicated at DIV49. Sample size: control n = 35 MEA wells, *SETD1A*^*+/*−^ n = 37 MEA wells from 4 independent batches (n = 25 from clone 1, n = 12 from clone 2). (D and E) Representative somatodendritic reconstructions of glutamatergic neurons and Sholl analyses in control and *SETD1A*^+/−^ networks at DIV 49 (n = 38 for control, n = 33 for *SETD1A*^+/−^ from clone 1). Red arrows indicate neurons reconstructed in the images. Scale bars, 40 μm. (F) Main morphological parameters for reconstruction of glutamatergic neurons at DIV49. (G–I) Representative voltage-clamp recordings of spontaneous inputs (sEPSCs) onto glutamatergic neurons at DIV49 and the corresponding quantitative analyses of sEPSC frequency (H) and amplitude (I). (J and K) Representative voltage-clamp recordings of correlated synaptic inputs (sEPSC bursts) in control and *SETD1A*^+/−^ glutamatergic neurons at DIV49 and the corresponding quantitative analyses (K). n = 24 for control, n = 17 for *SETD1A*^+/−^ clone 1 at DIV49. Data represent mean ± SEM. *p < 0.05, **p < 0.01, ***p < 0.001, two-way ANOVA with post hoc Bonferroni correction (E), unpaired Student’s t test (C, F) or Students’ T test with Bonferroni correction for multiple testing (H, I, K).

Our RNA-seq results clearly establish a link between *SETD1A* haploinsufficiency and glutamatergic synapse function. We therefore hypothesized that glutamatergic neurons might be prominent contributors to the network phenotype in *SETD1A*^+/−^ E/I cultures. To test this hypothesis, we set up homogeneous glutamatergic neuronal cultures (Figure 4A). We measured the glutamatergic neuronal network activity on MEA between DIV21 and DIV49 (Figure 4B). Consistent with the phenotype of E/I networks, *SETD1A*^+/−^ glutamatergic neuronal networks exhibited significantly higher burst activity and decreased burst duration and burst spike rate at DIV49. Global activity (i.e., the mean firing rate) did not differ between two groups (Figure 4C). Next we investigated whether, in glutamatergic cultures, there might be differences between control and *SETD1A*^*+/*−^ neurons at the single-cell level (Figures 4G–4K, S5A, and S5B; Table S3). Similar to the E/I cultures, we observed an increased sEPSC burst rate at the single-cell level, in particular at DIV49 (Figures 4J and 4K; Table S3). Taken together, our data show that E/I cultures and glutamatergic cultures are similarly affected when SETD1A expression is reduced. This was supported by the observation that, in glutamatergic neuronal networks, *SETD1A*^+/−^ neurons showed a similar increase in morphological complexity as in the E/I cultures (Figures 4D–4F and S5C–S5F). Finally, we performed a transcriptome analysis for glutamatergic cultures at DIV49. We identified, in total, 455 DEGs with 177 downregulated and 278 upregulated genes (Figures S5G and S5H). Notably, we again identified *SLITRK4* as a top hit (Figure S5H). Enrichment analysis detected GO terms such as “glutamatergic synapse,” “synapse organization,” “synapse assembly,” and many others related to synaptic function (Figure S5J). In disease association analysis, we found “bipolar disorder” and “SCZ” among the top hits (Figure S5I). Overall, *SETD1A*^+/−^ glutamatergic cultures recapitulate the network phenotype observed in the E/I cultures, indicating that SETD1A plays a major role in glutamatergic neurons.

### *SETD1A* haploinsufficiency leads to activation of the cAMP/PKA/CREB pathway

**Figure 5:**
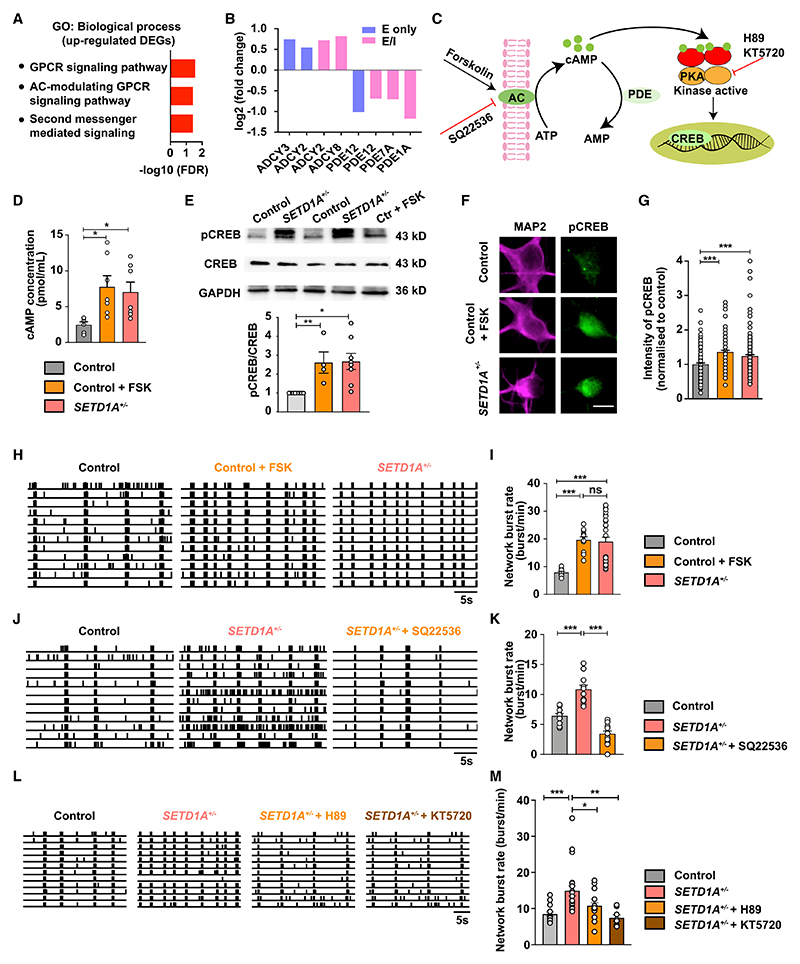
*SETD1A* haploinsufficiency leads to activation of the cAMP/PKA/CREB pathway (A) GO terms related to second messenger signaling associated with upregulated DEGs. (B) Change of mRNA levels of genes coding for adenylyl cyclase (AC) and phosphodiesterases. (C) Schematic of the cAMP/PKA/CREB pathway and drug targets at different steps. (D) cAMP concentrations for control (n = 6), control + 1 μM FSK (n = 7), and *SETD1A*^+/−^ cultures (n = 7 from clone 1). (E) Western blot of pCREB, CREB, and glyceraldehyde 3-phosphate dehydrogenase (GAPDH) for control (n = 8), control + 1 μM FSK (n = 4), and *SETD1A*^+/−^ cultures (n = 8 from clone 1). (F and G) Representative images showing pCREB expression and quantification of intensity of pCREB for control (n = 134 cells), control + 1 μM FSK (n = 96 cells),and *SETD1A*^+/−^ neurons (n = 156 cells, from 3 independent batches; n = 34 from clone 1 and n = 122 from clone 2). Scale bar, 10 μm. (H and I) Representative raster plot for 30 s recorded by MEA and quantification of network burst rate, showing the effect of the AC agonist FSK (1 μM) on control networks (sample size: control n = 10 wells, control + FSK n = 12 wells, *SETD1A*^+/−^ n = 28 wells [n = 6 from clone 1 and n = 22 from clone 2]). (J and K) Representative raster plot for 30 s recorded by MEA and quantification of network burst rate, showing the effect of the AC inhibitor SQ22536 (100 μM) on *SETD1A*^+/−^ E/I cultures (sample size: control n = 11 wells, *SETD1A*^+/−^ + SQ22536 n = 13 wells, *SETD1A*^+/−^ n = 13 wells from clone 2). (L and M) Representative raster plot for 30 s recorded by MEA and quantification of network burst rate, showing the effect of the PKA inhibitors H89 (2 μM) and KT5720 (1 μM) on *SETD1A*^+/−^ E/I cultures (sample size: control n = 15 wells, *SETD1A*^+/−^ + H89 n = 18 wells [n = 3 from clone 1 and n = 15 from clone 2], *SETD1A*^+/−^ + KT5820 n = 8 wells [n = 2 from clone 1 and n = 6 from clone 2], *SETD1A*^+/−^ n = 22 wells [n = 7 from clone 1 and n = 15 from clone 2]). Data represent mean ± SEM. *p < 0.05, **p < 0.01, ***p < 0.001, one-way ANOVA test and post hoc Bonferroni correction.

The changes in neuronal network activity observed in *SETD1A*^*+/*−^ cultures (E/I and glutamatergic cultures) suggest that pathways related to regulation of neuronal activity could be affected. Interestingly, in our transcriptome data, we found that the upregulated DEGs were enriched in several annotations related to second messenger signaling, such as “G protein-coupled receptor signaling pathway, coupled to cyclic nucleotide second messenger;” “second-messenger-mediated signaling;” and “adenylate cyclase-modulating G protein-coupled receptor signaling pathway” (Figure 5A; Table S4). In agreement with this finding, we found that multiple enzymes involved in synthesis and degradation of cAMP were dysregulated in E/I and glutamatergic cultures. Specifically, genes coding for proteins involved in cAMP production (*ADCY2, ADCY3*, and *ADCY8*) (Pieroni et al., 1995; Qiu et al., 2016; Wang and Storm, 2003) were significantly upregulated, whereas genes encoding for phosphodiesterases (*PDE12, PDE7A*, and *PDE1A*), important for cAMP degradation (Epstein, 2017; Evripioti et al., 2019; Keravis and Lugnier, 2012), were downregulated (Figure 5B). This strongly suggests that the cAMP pathway is hyperactive in *SETD1A*^+/−^ neurons. In support of this, in rodent hippocampal neuronal cultures, activating the cAMP pathway can lead to increased synchronized network activity (Angel-Chavez et al., 2015).

To test this hypothesis, we first measured the concentration of cAMP. As a positive control, we stimulated the cAMP pathway on control E/I cultures with 1 μM forskolin (FSK), a well-known adenylyl cyclase (AC) agonist and PKA/CREB pathway activator (Delghandi et al., 2005; Namkoong et al., 2009; Schiller et al., 2010; Figure 5C). In control E/I cultures, this FSK exposure resulted in a significantly increased concentration of cAMP (Figure 5D). Interestingly, without any treatment, in *SETD1A*^+/−^ cultures, the baseline cAMP level was significantly higher than in untreated control networks (Figures 5D and S2L). To confirm this, we measured the phosphorylation of cAMP response element binding protein (pCREB), a downstream signaling protein in the cAMP/PKA pathway (Tasken et al., 1993; Figure 5C). We performed western blotting and immunocytochemistry on DIV49 E/I cultures for anti-pCREB, an antibody detecting endogenous levels of CREB only when phosphorylated at serine^133^. In agreement with our previous findings, FSK significantly increased the levels of pCREB in control neurons (Figures 5E–5G). In addition, we found that the levels of pCREB in *SETD1A*^+/−^ neurons was significantly higher compared with control neurons (Figures 5E–5G). This result supports our hypothesis that the cAMP/PKA/CREB pathway exhibits increased activity in *SETD1A*^+/−^ neurons. Interestingly, ChIP-seq data of SETD1A in mice (Mukai et al., 2019) show that SETD1A binds to the promoter region of *ADCY2* and *ADCY8* (genes important for cAMP synthesis, up-regulated in our DEGs) and the enhancer region of *PDE1A* and *PDE7A* (genes important for cAMP degradation, down-regulated in our DEGs) (Figure 3B; Data S2). This suggests that these genes are direct target genes of SETD1A. We therefore infer that altered cAMP could be a primary change in *SETD1A*^+/−^ cultures.

To further investigate the link between enhanced cAMP activation and the neuronal network phenotype observed in *SETD1A*^+/−^ neurons, we bidirectionally modulated cAMP levels in control or *SETD1A*^*+/*−^ neurons from E/I cultures. Interestingly, we found that acute stimulation of DIV51 control neurons with 1 μM FSK for 1 h resulted in an increase in network burst rate, with a parallel reduction in inter-network burst interval (Figure 5H, 5I, and S6A–S6C), mimicking the network phenotype of *SETD1A*^+/−^ E/I cultures. In addition, burst spike rate and network burst duration were decreased, although the difference was not significant (Figure S6C). These results show that control networks treated with FSK resemble *SETD1A*^+/−^ neuronal networks. In a second set of experiments, we examined whether blocking cAMP using the AC inhibitor SQ22536 in *SETD1A*^+/−^ networks (starting from DIV42) would be sufficient to normalize the phenotype caused by *SETD1A* haploinsufficiency to control levels. Indeed, after 8 days of treatment with 100 μM SQ22536, we found that the major network parameters were normalized, including the network burst rate (Figures 5J, 5K, S7A, and S7B). We next investigated whether acute manipulation of the PKA pathway, a downstream target of cAMP, would be sufficient to normalize *SETD1A*^+/−^ network activity to control levels. Two independent chemical inhibitors of PKA, H89 and KT5720 (Murray, 2008; Song et al., 2015), were applied to *SETD1A*^+/−^ networks at DIV51 on MEA. A 1-h treatment with H89 (2 μM) or KT5720 (1 μM) in *SETD1A*^+/−^ networks was sufficient to normalize the major network parameters, including the network burst rate (Figures 5L, 5M, S7C–S7E, and S8A–S8D). Single-cell electrophysiological data showed that H89 treatment in *SETD1A*^+/−^ cultures significantly reduced rates of sEPSC burst events (Figures S8E–S8F). This indicates that blocking cAMP/PKA may directly influence synaptic function. Interestingly, in our *in vitro* model, cAMP/PKA blocking can rescue the network burst rate in SETD1A-deficient neuronal networks already at DIV21 (Figures S8A and S8B), a developmental stage without significant neuronal morphological alterations (Figures 2A and 2B), indicating that the rescue effect of the PKA inhibitor was mainly related to altered synaptic function. Finally, because of SETD1A’s function as a histone methyltransferase, the observed decrease in H3K4me3 in *SETD1A*^+/−^ neurons and the observation that the demethylation inhibitor ORY-1001 was sufficient to rescue the cognitive and circuitry deficits in the adult *Setd1a*^+/−^ rodent model (Mukai et al., 2019), we tested ORY-1001 in our human *in vitro* model. In accordance, exposure of *SETD1A*^+/−^ networks to 1 μM ORY-1001 was able to rescue the neuronal network phenotype (Figures S8G and S8H). Our results suggest that cAMP/PKA is one of the molecular pathways through which *SETD1A* haploinsufficiency leads to key neuronal network alteration.

## Discussion

Heterozygous LoF variants in *SETD1A*, with high penetrance for SCZ, provide an opportunity to investigate the neuronal dysfunction that might underlie the increased vulnerability to SCZ. Here we reveal crucial molecular signatures and neurodevelopmental abnormalities caused by *SETD1A* haploinsufficiency in human neurons. We show that *SETD1A* haploinsufficiency leads to altered neuronal network organization, to which changes in glutamatergic neuronal function might be one of the main contributors. We also identify increased cAMP/PKA activity as a molecular mechanism to the functional phenotype of *SETD1A*^*+/−*^ networks.

One major consistent characteristic of hiPSC-derived *SETD1A*^+/−^ networks is the increased network burst rate, which is reflected by an increase in synaptic connectivity at the single-cell level. Synchronized burst activity plays an important role during early brain development (Khazipov and Luhmann, 2006) and is considered to be a fundamental mechanism for information processing and, hence, relevant for perception, memory, and cognition (Lisman, 1997). Intriguingly, abnormalities in neural synchronization *in vivo*, which reflect neural circuit dysfunction, are suggested as one of the core pathophysiological mechanisms in SCZ (Ford et al., 2007; Uhlhaas and Singer, 2010). For example, increased oscillatory synchronization has been found to be correlated with positive symptoms of SCZ (Lee et al., 2006; Spencer et al., 2009), whereas increased and reduced oscillations are associated with negative symptoms (Lee et al., 2003; Uhlhaas and Singer, 2010). In the V1 of *Setd1a*^*+/−*^ mice, Hamm et al. (2020) identified reduced oscillations, providing circuit-level insight into the underlying neurobiology of sensory-processing abnormality seen in SCZ. In this regard, our human *in vitro* model and mouse model showed aberrant synchronized activity at different developmental stages, suggesting circuit disruption caused by *SETD1A* haploinsufficiency. Not only in *Setd1a*^+/−^ rodent models (Mukai et al., 2019), but also in our hiPSC derived *SETD1A*^+/−^ *in vitro* neuronal network, the demethylation inhibitor ORY-1001 could rescue cognitive and circuitry deficits (Mukai et al., 2019) or key parameters of aberrant neuronal network function. Further studies are required to better characterize the potential of ORY-1001 for clinical use.

Disrupted glutamatergic signaling plays a critical role in the pathogenesis of SCZ (Tsai and Coyle, 2002). On several levels, our data provide evidence supporting the idea that glutamatergic signaling is one of the main contributors to the network phenotype caused by *SETD1A* haploinsufficiency. First, through transcriptional profiling of *SETD1A*^+/−^ E/I cultures, we identified a genetic signature suggestive of perturbed function of glutamatergic signaling. Dysregulated genes include ones encoding for metabotropic glutamate receptors (*GRM4* and *GRM3*) and ionotropic glutamate receptors subunits (*GRIK3* and *GRIN2A*) as well as proteins involved in glutamatergic signaling (*SHISA6, SHISA7, CAMK4, PSD93*, and *VAMP1*). This is in line with the previous finding that target genes of SETD1A are mainly expressed in pyramidal neurons (Mukai et al., 2019). In particular, *SHISA6*, which is important for α-amino-3-hydroxy-5-methyl-4-isoxazole propionic acid (AMPA) receptor function, is strongly down-regulated, suggesting dysregulated AMPA receptor activity (Klaassen et al., 2016). In pyramidal neurons of cortical layer 2/3 (L2/3) of the mPFC in *Setd1a*^*+/−*^ mice, only excitatory synaptic transmission is altered, whereas inhibitory synaptic transmission remains unchanged (Nagahama et al., 2020). Second, in E/I networks, when GABAergic inputs were blocked by PTX, the phenotype of increased networks burst frequency still remained (Figure S2B). In addition, measurements from single-cell electro-physiology and MEA with homogeneous glutamatergic neurons show the same network signature as observed in E/I networks, indicating that glutamatergic neurons are one of the main driving factors in this phenotype. Third, transcriptomics data from E/I and glutamatergic cultures highlight a striking upregulation of *SLITRK4*, which is involved in neurite outgrowth and synaptogenesis, especially in glutamatergic synapse formation (Yim et al., 2013). Although our data gave no indication of a prominent difference in synapse density between genotypes, increased frequencies of miniature EPSCs and the more extensive dendritic organization of *SETD1A*^+/−^ neurons indicate elevated total numbers of synapses per *SETD1A*^+/−^ neuron. This increase in synaptic connectivity may be related at least partially to the increased SLITRK4 in *SETD1A*^+/−^ neurons. It has been shown that variants in *SLITRK4* are associated with neuropsychiatric disorders, such as SCZ (Kang et al., 2016). *SLITRK4* has also been identified as a DEG in *Setd1a*^*+/*−^ mice (Mukai et al., 2019), emphasizing its important role as a downstream target of SETD1A across species. Indeed, our data show that *SETD1A* haploinsufficiency leads to increased dendritic complexity in human glutamatergic neurons. This morphological phenotype was not detected in the *SETD1A*^+/−^ mouse model of Mukai et al. (2019), where pyramidal neurons in mature prefrontal cortical networks showed unchanged dendritic arborization. This difference can potentially be explained by the differences in the assessed developmental stages or species-related differences in SETD1A function. These results imply that SETD1A plays an essential role in the function of glutamatergic signaling. However, dysfunction of GABAergic signaling might be involved in the network phenotype as well. Our results show that *SETD1A* haploinsufficiency leads to increased dendritic complexity of GABAergic neurons, which likely affects the input connectivity and signal integration of these neurons. Further experiments are needed to clearly dissect the role of SETD1A in GABAergic neurons.

Our data identify several molecular pathways that may contribute to the increased network burst frequency in *SETD1A*^+/−^ networks. In particular, our transcriptomic data indicated that *SETD1A* haploinsufficiency leads to increased cAMP/PKA, which has been shown to enhance synaptic strength through different aspects, such as by modulating different ion channels (Anderson et al., 2000; Beaumont and Zucker, 2000; Connors et al., 2008; Gutierrez-Castellanos et al., 2017; Hell’ et al., 1995; van der Horst et al., 2020; Renner et al., 2017) and increasing neurite outgrowth (Aglah et al., 2008; Chu et al., 2006; Wan et al., 2011). Intriguingly, a recent study demonstrated that cAMP/PKA induces calcium influx through voltage-gated calcium channels, which pushes the neuronal network toward large-scale and synchronized burst activity (Thornquist et al., 2021). Our calcium imaging data showed increased synchronized activity in *SETD1A*^+/−^ networks, suggesting that there could be a similar cAMP-triggered mechanism in our model.

cAMP signaling has been implicated in neurodevelopmental and neuropsychiatric disorders, including SCZ (Funk et al., 2012; Millar et al., 2005; Muly, 2002; Turetsky and Moberg, 2009; Vacic et al., 2011; Wang et al., 2018), bipolar disorder (Chang et al., 2003; Ren et al., 2014), fragile X syndrome (Berry-Kravis et al., 1995; Berry-Kravis and Huttenlocher, 1992; Kelley et al., 2007), and autism (Kelley et al., 2008; Zamarbide et al., 2019). For example, in individuals with bipolar disorder, cAMP/PKA signaling is upregulated (Chang et al., 2003), whereas in fragile X syndrome, cAMP/PKA signaling is decreased (Berry-Kravis et al., 1995). These findings emphasize the essential role of balanced cAMP activity in normal brain function, making it an appealing therapeutic target. Interestingly, our results show that, on the network level, blocking the PKA pathway and inhibiting AC can rescue the phenotype. However, we cannot rule out the possibility that other downstream effectors of cAMP, such as EPAC (a guanine nucleotide exchange factor) and cyclic-nucleotide-gated ion channels (Sassone-Corsi, 2012), are also partly involved in the phenotype caused by *SETD1A* haploinsufficiency. Because cAMP/PKA signaling functions in various cells types, therapeutic intervention of its activity needs further clarification of its cell-type-specific roles. In this context, future studies should comprehensively investigate whether upregulated cAMP/PKA signaling caused by *SETD1A* haploinsufficiency also applies to other brain regions or other cell types related to SCZ or neurodevelopmental disorders, such as serotonergic (Carvajal-Oliveros and Campusano, 2021) or dopaminergic neurons (Eells, 2003) and glial cells (Blanco-Suárez et al., 2017).

### Limitations of the study

One limitation of the current study is that the transcriptomic data were collected from bulk RNA-seq. Because of the lower percentage of GABAergic neurons in our E/I neuronal network model, the power to detect changes in pre-synaptic GABAergic signaling might be underestimated. Additional studies are needed to investigate the specific role of SETD1A in GABAergic neuronal function. Another limitation is that the accelerated neuronal differentiation approach we employed for our current human neuronal *in vitro* model skips certain early developmental stages, such as the neuro-progenitor cell stage, of neuronal networks, and it cannot capture the cellular complexity of fully mature cortical networks. However, the defined composition of human neuronal networks allows assessment of core molecular and cellular mechanisms related to *SETD1A* haploinsufficiency in postmitotic neurons.

Our data suggest glutamatergic synaptic dysfunction is one of the potential pathogenic mechanisms of *SETD1A* haploinsufficiency-associated disorders, and we identified cAMP/PKA dysregulation as underlying mechanisms responsible for the altered network phenotype in *SETD1A*^*+/*−^ cultures. Future studies using rodent models and human *in vitro* neuronal models are required to further explore the effect and therapeutic potential of cAMP/PKA in *SETD1A*-associated disorders.

## Star★Methods

Detailed methods are provided in the online version of this paper and include the following:

● KEY RESOURCES TABLE
● RESOURCE AVAILABILITY
  ○ Lead contact
  ○ Materials availability
  ○ Data and code availability
● EXPERIMENTAL MODEL AND SUBJECT DETAILS
  ○ Animals
  ○ Human iPSC lines
  ○ Neuronal differentiation
● METHODS DETAILS
  ○ CRISPR/Cas9 editing of *SETD1A*
  ○ Immunocytochemistry
  ○ Western Blot
  ○ Neuron reconstruction
  ○ MEA recordings and analysis
  ○ Chemicals
  ○ Pharmacological experiment
  ○ cAMP ELISA kit
  ○ Whole cell patch clamp
  ○ Calcium imaging
  ○ RNA sequencing
  ○ RNA-seq data processing
  ○ Differential expression (DE) analysis and over-representation test
● QUANTIFICATION AND STATISTICAL ANALYSIS

## Star ★ Methods

### Key Resources Table

**Table:** 

REAGENT or RESOURCE		SOURCE	IDENTIFIER		
Antibodies					
Rabbit anti-MAP2		Abcam	Ab32454; RRID: AB_776174		
Rabbit anti-VGAT		Synaptic Systems	131013; RRID: AB_2189938		
Rabbit anti-Phospho-CREB		Cell Signaling	9198; RRID: AB_2561044		
Rabbit anti-CREB		Cell Signaling	9197S; RRID: AB_331277		
Rabbit anti-GAPDH		Cell Signaling	2118S; RRID: AB_561053		
Rabbit anti-H3K4me3		Abcam	Ab32356; RRID: AB_732924		
Rabbit anti-GABA		Sigma-Aldrich	A0310; RRID: AB_476667		
Mouse anti-SET1A		Santa Cruz	SC-515590; RRID: N/A		
Mouse anti-γ-tubulin		Sigma-Aldrich	T5326; RRID: AB_532292		
Mouse anti-Gephyrin		Synaptic Systems	147011; RRID: AB_887717		
Guinea pig anti-Synapsin 1/2		Synaptic Systems	106004; RRID: AB_1106784		
Goat anti-rabbit IgG		Invitrogen	G-21234; RRID: AB_2536530		
Goat anti-mouse IgG		Jackson Immuno Research Laboratories	115-035-062; RRID: AB_2338504		
Goat-anti rabbit Alexa 568		Invitrogen	A11036; RRID: AB_10563566		
Goat-anti-mouse Alexa 488		Invitrogen	A11029; RRID: AB_2534088		
Goat anti-guinea pig Alexa Fluor 568		Invitrogen	A11075; RRID: AB_2534119		
Goat anti-guinea pig Alexa Fluor 647		Invitrogen	A21450; RRID: AB_141882		
Bacterial and virus strains					
AAV2-hSyn-mCherry		UNC Vector Core	AV5033E		
Chemicals, peptides, and recombinant proteins					
Accutase		GIBCO	A11105-01		
B27 Supplement		GIBCO	0080085SA		
Cytosine β-D-arabinofuranoside (Ara-C)		Sigma-Aldrich	C1768-100MG		
BDNF, human recombinant		Promokine	C66212		
DAKO fluorescent mounting medium		DAKO	S3023		
DMEM/F12		GIBCO	11320–074		
Doxycycline		Sigma-Aldrich	D9891-5G		
DPBS		GIBCO	14190–094		
E8 Basal Medium		GIBCO	A1517001		
Fetal Bovine Serum (FBS)		Sigma-Aldrich	F2442-500ML		
Fluo-8-AM		Abcam	Ab142773		
Forskolin		Sigma-Aldrich	F6886		
G418		Sigma-Aldrich	4727878001		
GlutaMAX		GIBCO	35050061		
Hoechst 33342		Thermo-Fisher	H3570 N/A		
H89		Tocris	2910		
KT5720		Tocris	1283		
Matrigel		Corning	356237		
Non-essential amino acid solution NEAA		Sigma-Aldrich	M7145		
N2		GIBCO	17502–048		
Neurobasal Medium		GIBCO	21103–049		
NT-3, human recombinant		Promokine	C66425		
ORY1001	SelleckChem			RG6016	
Penicillin/Streptomycin	Sigma-Aldrich			P4333	
Poly-L-ornithine hydrobromide (PLO)	Sigma-Aldrich			P3655-10MG	
Primocin	Invivogen			ANT-PM-05	
Puromycin	Invivogen			ANT-PR-1	
Picrotoxin	Tocris			1128	
Recombinant Human Laminin LN521	Biolamina			2021–21	
RevitaCell	Thermo-Fisher			A2644501	
ReLeSR	Stem Cell Technologies			058072	
SQ22536	Sigma-Aldrich			S153	
Triton X-100	Sigma-Aldrich			9002-93-1	
Critical commercial assays					
24-well MEA system	Multichannel Systems, MCS GmbH, Reutlingen, Germany			N/A	
Zeiss Axio Imager Z1 Olympus	Zeiss			https://www.zeiss.com/	
Multiclamp 700B amplifier	Molecular Devices, Wokingham, United Kingdom			https://www.moleculardevices.com/	
cAMP ELISA kit	Abcam			Ab133051	
BCA protein assay kit	Thermo-Fisher			23225	
iMark™ Microplate Absorbance Reader	Bio-Rad			N/A	
Deposited data					
RNA-seq	This paper			GEO: GSE180648	
Code	This paper			Zenodo: https://doi.org/10.5281/zenodo.6409947	
Experimental models: Human iPSC lines					
CTR-WTC	Corielll Institute			https://www.coriell.org/0/Sections/Search/Sample_Detail.aspx?Ref=GM25256&Product=CC	
WTC-CRISPR-clone 1	Generated in this study			N/A	
WTC-CRISPR-clone 2	Generated in this study			N/A	
CTR-WTC-Ngn2/rtTA	Generated in this study			N/A	
CTR-WTC-Ascl1/rtTA	Generated in this study			N/A	
WTC-CRISPR-clone 1- Ngn2/rtTA	Generated in this study			N/A	
WTC-CRISPR-clone 1- Ascl1/rtTA	Generated in this study			N/A	
WTC-CRISPR-clone 2- Ngn2/rtTA	Generated in this study			N/A	
WTC-CRISPR-clone 2- Ascl1/rtTA	Generated in this study			N/A	
CTR-409b2	Yamanaka, Shinya / RIKEN BRC Generated			HPS0076; 409B2; RRID: CVCL_K092	
409b2-CRISPR	Generated in this study			N/A	
CTR-409b2- Ngn2/rtTA	Generated in this study			N/A	
CTR-409b2- Ascl1/rtTA	Generated in this study			N/A	
409b2-CRISPR- Ngn2/rtTA	Generated in this study			N/A	
409b2-CRISPR- Ascl1/rtTA	Generated in this study			N/A	
Experimental models: Organisms/strains					
Wistar WT Rat (Dissociated astrocytes)	Charles River			N/A	
Recombinant DNA					
Lentivirus psPAX2 packaging vector Lentivirus	psPAX2 was a gift from Didier Trono			RRID: Addgene_12260; http://addgene.org/12260	
Lentivirus VSVG envelope glycoprotein vector pMD2-G	pMD2.G was a gift from Didier Trono			RRID: Addgene_12259; http://addgene.org/12259	
pSpCas9(BB)-2A-Puro (PX459)		Addgene		62988	
pLVX-EF1α-(Tet-On-Advanced)-IRES-G418(R) lentiviral vector		Mossink et al., 2021		N/A	
pLVX-(TRE-thight)-(MOUSE) Ngn2-PGK-Puromycin(R) lentiviral vector		Mossink et al., 2021		N/A	
pLV[TetOn]-Puro-TRE > mAscl1 lentiviral vector		Mossink et al., 2021		N/A	
Software and algorithms					
GraphPad Prism		GraphPad software		https://www.graphpad.com/scientific-software/prism/; RRID:SCR_002798	
Fiji		National Institutes of Health		https://imagej.nih.gov/ij/download.html RRID: SCR_003070	
ClampfitV 10.2		Molecular Devices, LLC., CA, USA		RRID: SCR_011323	
MATLAB 2014b		Mathworks		RRID: SCR_001622	
Multiwell Analyzer		Multichannel Systems, MCS GmbH, Reutlingen, Germany		N/A	
Neurolucida 360		MBF-Bioscience, Williston, ND, USA		RRID: SCR_016788	
Image lab		Bio-Rad		https://www.bio-rad.com/en-nl/product/image-lab-software/	

### Resource Availability

#### Lead contact

Further information and requests for resources and reagents should be directed to and will be fulfilled by the lead contact: Dirk Schubert d.schubert@donders.ru.nl.

#### Materials availability

This study did not generate novel unique reagents except hiPSC lines. The hiPSC lines generated in this study can be made available on request, but we may require a payment and/or a completed Materials Transfer Agreement if there is a potential for commercial application.

### Experimental Model and Subject Details

#### Animals

For the dissection and culturing of rat astrocytes, pregnant WT Wistar rats from Charles River were sacrificed after which embryos (E18) were removed for generating primary cultures. Animal experiments were conducted in conformity with the Animal Care Committee of the Radboud University Nijmegen Medical Center, the Netherlands, under DEC application number 2015–0038, and conform to the guidelines of the Dutch Council for Animal Care and the European Communities Council Directive 2010/63/EU.

#### Human iPSC lines

hiPSCs (WTC) used in this study were obtained from reprogrammed fibroblasts of one healthy male doner, 30-year old (Miyaoka et al., 2014). 409b2 hiPSCs were derived from fibroblasts of a 36-year-old female. To investigate the role of SETD1A, CRISPR/Cas9 was used to induce loss-of-function mutation of *SETD1A* into these two healthy control lines. hiPSCs were cultured on a 6-well plate pre-coated with 1:15 (diluted in DMEM/F12 medium) Matrigel (Corning, #356237) in Essential 8 Flex medium (Thermo Fisher Scientific) supplemented with primocin (0.1 g/mL, Invitrogen) at 37°C/5% CO_2_.

To make *Ngn2* or *Ascl1*-stable hiPSC lines, hiPSCs were infected with *Ascl1* or *Ngn2* and *rtTA* lentivirus (The transfer vector used for the rtTA lentivirus is pLVX-EF1α-(Tet-On-Advanced)-IRES-G418(R); The transfer vector used for the Ngn2 lentivirus is pLVX-(TRE-thight)-(MOUSE) Ngn2-PGK-Puromycin(R); The transfer vector used for the Ascl1 lentivirus is pLV[TetOn]-Puro-TRE > mAscl1. All the plasmids are available upon request). Medium was supplemented with puromycin (0.5 g/mL) and G418 (50 g/mL). Medium was refreshed every 2–3 days and hiPSCs were passaged twice per week using an enzyme-free reagent (ReLeSR, Stem Cell Technologies).

#### Neuronal differentiation

The neuronal differentiation protocol used in this article was previously described (Mossink et al., 2021). Glutamatergic neurons were either cultured alone or in coculture with GABAergic neurons. When cocultured, GABAergic neurons were plated at days *in vitro* (DIV) 0 and transduced with AAV2-hSyn-mCherry (UNC Vector Core) for visualization. After 4 h incubation, cultures were washed twice with DMEM/F12 (Thermo Fisher Scientific) after which glutamatergic neurons were plated into the well. hiPSCs were plated in E8 flex supplemented with doxycycline (4 μg/mL), Revitacell (1:100, Thermo Fisher Scientific) and Forskolin (10 μM, Sigma). At DIV 1 cultures were switched to DMEM/F12 containing Forskolin, N2 (1:100, Thermo Fisher Scientific), non-essential amino acids (1:100, Sigma), primocin (0.1 μg/mL, Invivogen), NT3 (10 ng/mL, PromoCell), BDNF (10 ng/mL, PromoCell), and doxycycline (4 μg/mL). To support neuronal maturation, freshly prepared rat astrocytes were added to the culture in a 1:1 ratio at DIV 2. At DIV 3 the medium was changed to Neurobasal medium (Thermo Fisher Scientific) supplemented with Forskolin (10 μM, Sigma), B-27 (Thermo Fisher Scientific), GlutaMAX (Thermo Fisher Scientific), primocin (0.1 μg/mL), NT3 (10 ng/mL), BDNF (10 ng/mL), and doxycycline (4 μg/mL). To remove the proliferating cells from the culture, cytosine-b-D-arabinofuranoside (Ara-C, 2 μM, Sigma) was added to the medium at DIV 3. From DIV 6 onwards half of the medium was refreshed three times a week. From DIV 10 onwards, the medium was additionally supplemented with 2.5% FBS (Sigma) to support astrocyte viability. After DIV 13, Forskolin and doxycycline were removed from the medium. Cultures were kept at least until DIV49.

### Methods Details

#### CRISPR/Cas9 editing of *SETD1A*

CRISPR/Cas9 technology was used to create a heterozygous indel mutation in exon 7 of *SETD1A* in a healthy hiPSC line derived from a male, 30 years old (Mandegar et al., 2016). In brief, sgRNAs were designed to specifically target *SETD1A* (GTCCTTGGGGC CAGAGATAC AGG), and then cloned into pSpCas9(BB)-2A-Puro (PX459) V2.0 (Addgene #62988). Single-cell suspension of hiPSCs was nucleofected with 5 μg of the generated SpCas9-sgRNA plasmid using the P3 Primary Cell 4D-Nucleofector Kit (Lonza, #V4XP-3024) in combination with the 4D Nucleofector Unit X (Lonza, #AAF-1002X), program CA-137. After nucleofection, cells were resuspended in E8 Flex supplemented with Revitacell and seeded on Matrigel pre-coated plates. One day after the nucleofection, 0.5 μg/mL puromycin was added for 24 h for selection. Puromycin-resistant colonies were manually picked and Sanger Sequencing was performed to ensure heterozygous editing of exon 7. Two positive clones were selected for further characterization. Cells were tested routinely as mycoplasma-negative. The expression of pluripotency markers OCT4, NANOG, SSEA4 and TRA1-81 were detected with immunocytochemistry. Karyotyping was performed as a service by the Diagnostics Department at Radboud University Medical Center. Potential off-target sites were checked by sanger sequencing.

#### Immunocytochemistry

Cells seeded on coverslips were fixed with 4% paraformaldehyde supplemented with 4% sucrose for 15 min at room temperature, followed by permeabilization with 0.2% Triton for 10 min. Nonspecific binding sites were blocked by incubation in 5% normal goat serum for 1 h at room temperature. Cells were incubated with primary antibodies overnight at 4°C. The second day, secondary antibodies, conjugated to Alexa-fluorochromes, were added and incubated for 1 h at room temperature. Hoechst 33342 was used to stain the nucleus before cells were mounted with fluorescent mounting medium (DAKO, S3023). The following primary antibodies were used: Rabbit anti-MAP2 (1:1000, Abcam, ab32454); Mouse anti-Gephyrin (1:1000, Synaptic Systems 147011); Rabbit anti-VGAT (1:500, Synaptic systems 131013); Guinea pig anti-Synapsin 1/2 (1:1000, Synaptic Systems 106004); Mouse anti-Homer1 (1:500, Synaptic Systems 160011); Rabbit anti-Phospho-CREB (1:500, Cell Signaling 87G3 9198); Rabbit anti-H3K4me3 (1:500, Abcam, ab32356); Rabbit anti-GABA (1:500, Sigma, A0310). Secondary antibodies that were used are: Goat-anti rabbit Alexa 568 (1:1000, Invitrogen, A11036); Goat-anti-mouse Alexa 488 (1:1000, Invitrogen, A11029); Goat anti-guinea pig Alexa Fluor 568 (1:2000, Invitrogen, A11075); Goat anti-guinea pig Alexa Fluor 647 (1:1000, Invitrogen, A21450). Cells were imaged at 63x magnification using the Zeiss Axio Imager Z1 equipped with ApoTome. Fluorescent images were analyzed using FIJI software (Schindelin et al., 2012). Synapse puncta were counted manually and normalized to the length of the dendritic branch where they reside.

#### Western Blot

To lyse the cells, medium was removed and the well was washed with 2 mL ice-cold PBS before 100 μL lysis buffer were applied (RIPA buffer supplemented with PhosSTOP; Roche) and protease inhibitors (complete Mini, EDTA free; Roche). Before blotting the protein, concentration was determined by means of a Pierce™ BCA protein assay (Thermo Scientific 23225). For each sample, the same amount of protein around 15 μg was loaded and separated by SDS-PAGE. Depending on the primary antibody, separated proteins were transferred to PVDF membrane (BioRad). Primary antibodies were used are: Mouse anti-SET1A (1: 500, Santa Cruz 515590); Mouse anti-γ-tubulin (1:1000, Sigma T5326); Rabbit anti-CREB (1:1000, Cell signaling, 9197); Rabbit anti-Phospho-CREB (1:1000, Cell signaling, 9198); Rabbit anti-GAPDH (1:1000, Cell signaling, 2118). For visualization horseradish peroxidase-conjugated secondary antibodies were used: Goat anti-mouse IgG (1:20000, Jackson Immuno Research Laboratories 115-035-062), Goat anti-rabbit IgG (1:10000, Invitrogen, G21234).

#### Neuron reconstruction

Reconstruction was performed using Neurolucida 360 (Version 2017.01.4, Microbrightfield Bioscience). Neurons were fixed and labeled with MAP2 antibody. To distinguish GABAergic neurons from glutamatergic neurons, we infected GABAergic neurons with AAV2-hSyn-mCherry for (UNC Vector Core) visualization. We chose two time points to fix the neurons: DIV21 and DIV49. This allows us to compare the morphological phenotype at different developmental stages. Fluorescent images of MAP2-labelled neurons were taken at 20x magnification using Zeiss Axio Imager Z1 equipped with ApoTome. The images were stitched using Fiji 2018 software with the stitching plugin and followed by reconstruction using Neurolucida 360 (Version 2017.01.4, Microbrightfield Bioscience). The 3-dimensional reconstructions and quantitative morphometrical analyses focused on the somatodendritic organization of the neurons. The axon was excluded based on its long, thin properties and far-reaching projections. Neurons that had at least two primary dendrites were selected for reconstruction and further analysis. For morphometrical analysis, we determined soma size, number of primary dendrites, dendritic nodes and ends and total or mean dendritic length as well as covered surface by dendritic trees. In addition, we also investigated dendritic complexity by performing Sholl analysis. Sholl profile was obtained by applying a series of concentric circles at 20 μm interval from the soma center, subsequently, dendritic length, number of intersections and number of nodes of the neurons were measured for each distance interval.

#### MEA recordings and analysis

All recordings were performed using the 24-wells MEA system (Multichannel Systems, MCS GmbH, Reutlingen, Germany). Recordings and analysis were performed according to previous published protocols (Frega et al., 2019). Briefly, spontaneous electrophysiological activity of hiPSC-derived neuronal networks cultured on MEA was recorded for 10 min in a recording chamber which was constantly maintained at 37°C with 95% O_2_ and 5% CO_2_. Before recording, cultures on MEA were allowed to adapt for 10 min in the recording chamber. The recording was sampled at 10 kHz, and filtered with a high-pass filter with a 100 Hz cut-off frequency and a low-pass filter with a 3500 Hz cut-off frequency. The spike detection threshold was set at ± 4.5 standard deviations. Spike, burst and network burst detection was performed by a built-in algorithm in Mulitwell Analzer software (Multichannel Systems), and a custommade MATLAB (The Mathworks, Natrick) code to extract parameters characterizing network activity. Mean firing rate (MFR) was calculated as the average of the spike frequency of all channels across one MEA well. From the burst detection, the number of bursting channels (above threshold 0.4 burst/s and at least 5 spikes in burst with a minimal inter-burst-interval of 100 ms) was determined. A network burst was defined when at least 50% of the channels in one well exhibited a synchronous burst.

#### Chemicals

All reagents were prepared fresh into concentrated stocks, and stored frozen at –20°C. The following compounds were used in pharmacological experiments: Picrotoxin (100 mM in DMSO, Tocris 1128); Forskolin (12 mM in DMSO, Sigma F6886); SQ22536 (50 mM in DMSO, Sigma S153); H89 (5 mM in MQ, Tocris 2910); KT5720 (1 mM in DMSO, Tocris 1283). For all experiment on MEA, before adding chemical to the cultures, an aliquot of the concentrated stock was first diluted in DPBS at room temperature. Then, the appropriate amount of working dilution was added directly to wells on the MEA and mixing was primarily through diffusion into the (500 μL) cell culture medium.

#### Pharmacological experiment

Control and *SETD1A*^+/-^ networks on MEA were treated with Picrotoxin (PTX, 100 μM), Forskolin (1 μM), H89 (2 μM) and KT5720 (1 μM) at DIV49 or DIV51, and SQ22536 (100 μM) at DIV42 after a 20 min recording of spontaneous activity. Then the recording was stopped temporarily, and the compounds were added to the MEA. We recorded neuronal network activity for 10 min after 5 min treatment of PTX, 60 min treatment of Forskolin, KT5720, H89 and 8 days treatment of SQ22536. ORY-1001 (1 μM) was added to the *SETD1A*^+/-^ networks since DIV10 until DIV51.

#### cAMP ELISA kit

This experiment was performed according to the data sheet of the cAMP ELISA kit (Abcam, ab133051).

#### Whole cell patch clamp

Whole cell patch clamp was performed as previously described (Mossink et al., 2021). Coverslips were placed in the recording chamber of the electrophysiological setup, continuously perfused with oxygenated (95% O_2_/5% CO_2_) ACSF at 32°C containing (in mM) 124 NaCl, 1.25 NaH_2_PO4, 3 KCl, 26 NaHCO_3_, 11 Glucose, 2 CaCl_2_, 1 MgCl_2_. Patch pipettes with filament (ID 0.86 mm, OD1.05 mm, resistance 6–8 MΩ) were pulled from borosilicate glass (Science Products GmbH, Hofheim, Germany) using a Narishige PC-10 micropipette puller (Narishige, London, UK). For all recordings of intrinsic properties and spontaneous activity and mPSC activity, a potassium-based intracellular solution containing (in mM) 130 K-Gluconate, 5 KCl, 10 HEPES, 2.5 MgCl_2_, 4 Na_2_-ATP, 0.4 Na_3_-ATP, 10 Na-phosphocreatine and 0.6 EGTA was used, with a pH of 7.2 and osmolality of 290 mOsmol/L. Resting membrane potential (Vrmp) was measured immediately after generation of a whole-cell configuration. Further analysis of active and passive membrane properties was conducted at a holding potential of –60 mV. Passive membrane properties were determined via voltage steps of –10 mV. Active intrinsic properties were measured with a stepwise current injection protocol. Spontaneous post-synaptic currents (sPSCs) and miniature postsynaptic currents (mPSCs) were measured by 10 min continuous recording at a holding potential (V_h_) of –60 mV. In our in-vitro cultures GABAergic spontaneous and miniature events are very sparse (Mossink et al., 2021), thus detected PSCs were considered as mainly reflecting glutamatergic excitatory postsynaptic currents (EPSCs). sEPSC burst inputs were manually counted using clampfit 10.7. sEPSCs were grouped as a burst if at least 3 consecutive events occurred within 50 ms, with at least one of these events showing an amplitude above 100 pA. For the recording of mEPSCs, 1 μM TTX was added to the recording medium. Recordings were conducted at either DIV 21 and DIV 49 (intrinsic properties and sEPSCs) or only DIV 49 (mEPSCs). Cells were visualized with an Olympus BX51WI upright microscope (Olympus Life Science, PA, USA), equipped with a DAGE-MTI IR-1000E (DAGE-MTI, IN, USA) camera) and a CoolLED PE-200 LED system (Scientifica, Sussex, UK) which aided in fluorescent identification of GABAergic neurons. A Digidata 1440A digitizer and a Multiclamp 700B amplifier (Molecular Devices) were used for data acquisition. Sampling rate was set at 20 kHz (intrinsic properties) or 10 kHz (sEPSCs and mEPSCs) and a lowpass 1 kHz filter was used during recording. Recordings were not corrected for liquid junction potential (±10 mV). Recordings were discarded if series resistance reached >25 MΩ or dropped below a 10:0 ratio of membrane resistance to series resistance. Intrinsic electrophysiological properties were analyzed using Clampfit 11.2 (molecular devices, CA, USA), and sEPSCs were analyzed using MiniAnalysis 6.0.2 (Synaptosoft Inc, GA, USA) as previously described (Mossink et al., 2021). Regarding the analysis of the intrinsic properties: In brief, the adaptation ratio was defined as the Δt action potential 8–9/Δt action potential 2–3. The afterhyperpolarization time was defined as the time from which the repolarization phase reaches the threshold potential to the time at which the most hyperpolarized potential was reached. Action potential half-time was calculated as the time to reach 50% ΔmV of the action potential to the AHP peak.

#### Calcium imaging

For calcium imaging, cultures were incubated with 4 μg/mL Fluo-8-AM for 30 min at 37°C. After incubation, we removed the excess dye by washing the cells 3 times with HHBS. The cells were then left in culture medium to recover for 15 min. To image the cells, we placed them under the microscope (SliceScope Pro 2000, Scientifica). We continuously perfused the recording chamber with oxygenated (95% O_2_/ 5% CO_2_) and artificial cerebrospinal fluid (ACSF) that was composed of (in mM) 124 NaCl, 3 KCl, 1.25 NaH_2_PO_4_, 2 CaCl_2_, 1 MgCl_2_, 26 NaHCO_3_, 10 Glucose and heated to 37°C. Imaging was performed using a sCMOS camera (Prime BSI Express, Teledyne Photometrics) controlled by Micro-Manager acquisition software (NIH). Fluo-8-AM in the cells was excited at 470 nm by LED (KSL470, Rapp OptoElectronic). We recorded the cells for 2 min with frame rate of 10 Hz. After recording, we analyzed the video using MATLAB (The Math Works, Inc. MATLAB. Version 2020b) with a home-made script based on Sun et al. (Sun and Su üdhof, 2020). Circular ROIs were chosen by hand at the center of the soma with a diameter of 10 μm. We obtained the fluorescent change over time which is defined as ΔF/F = (F-F_0_)/F_0._ Furthermore, the decay of baseline intensity due to bleaching was corrected by exponential fitting to the baseline. Additionally, the traces of each location were analyzed for a synchronous firing rate among the selected ROIs to determine network patterns.

#### RNA sequencing

Cells were harvested on DIV49 of neuronal differentiation. For RNA-seq, the prepared samples were sequenced on an Illumina NovaSeq SP platform at an average depth of ~50 million reads per sample using a read length of 100 base pairs and an insert size of 350 base pairs. Three biological replicates of control and *SETD1A*^+/-^ E/I networks and glutamatergic networks respectively (12 samples in total) using the NucleoSpin RNA isolation kit (Machery Nagel, 740955.250) according to the manufacturer’s instructions. RNA yield was quantified with a NanoDrop Spectrophotometer (NanoDrop Technologies, Wilmington, DE, USA) and RNA integrity was assessed with Bioanalyzer 2100 RNA 6000 Nano Kit (Agilent Technologies, Santa Clara, CA, USA). All samples had an RNA Integrity Number (RIN) > 9. Library preparation and paired-end RNA-sequencing were carried out at the Norwegian High-Throughput Sequencing Center (www.sequencing.uio.no). Briefly, libraries were prepared with the TruSeq Stranded mRNA kit from Illumina which involves Poly-A purification to capture coding as well as several non-coding RNAs. The prepared samples were then sequenced on an Illumina NovaSeq SP platform.

#### RNA-seq data processing

Raw sequencing reads were quality assessed with FastQC (Babraham Institute). To pass the initial QC check, the average Phred score of each base position across all reads had to be at least 30. Reads were further processed by cutting individual low-quality bases and removing adapter and other Illumina-specific sequences with Trimmomatic V0.32 using default parameters (Bolger et al., 2014). The trimming process may result in some reads being discarded and their mates thereby unpaired, therefore only reads that remained paired after trimming were used for downstream analyses. Since the cultures contained both hiPSC-derived neurons as well as rat astrocytes which were added to support neuronal maturation, sequencing reads were separated according to their species of origin using the *in silico* RNA-seq read sorting tool Sargasso (Qiu et al., 2018), which was able to successfully eliminate sequencing reads stemming from rat astrocytes (Figure S9). To quantify gene expression levels, reads mapped by Sargasso were summarized at the gene level using feature Counts (Liao et al., 2014) guided by ENSEMBL annotations.

#### Differential expression (DE) analysis and over-representation test

To evaluate the species separation performance of Sargasso, bioinformatical estimation of cell type abundances (deconvolution) was carried out with CIBERSORTx (Newman et al., 2019) using expression signatures for human neurons and rodent astrocytes (Zhang et al., 2016) (Figure S9). Before conducting the DE analyses, genes with very low to zero expression were removed by filtering out any gene with ≤ 1 counts per million (CPM) in 3 or more samples (the smallest group size). DE analysis was performed using the statistical R package DESeq2 (Love et al., 2014), which provides methods to test for differentially expressed genes by use of negative binomial generalized models. The DESeq2 workflow begins by taking raw read count data as input and applies an internal normalization method that corrects for sequencing depth and RNA composition. The standard DE analysis consists of size factor estimation, dispersion estimation, and model fitting, as well as an independent filtering step that optimizes the number of significant DE genes. After the pre-filtering and independent filtering steps, a total of 14,144 genes were retained and examined in the DE analyses. A DE gene was considered significant if the FDR was <0.05. Gene Ontology (GO) enrichment tests of significant DE gene sets were conducted with the over-representation analysis tool clusterProfile (Yu et al., 2012) using the enrichGO function. A GO term was considered significantly enriched if the FDR was <0.05. Disease association analysis was performed with the R package disgenet2r (Piñero et al., 2020) using both the CURATED database, which includes more than 10,000 somatic and mental disorders, and the PSYGENET, which includes 109 mental diseases. In both cases, FDR<0.05 was used as the threshold to determine significant disease association.

### Quantification and Statistical Analysis

The statistical analysis of the data were performed using GraphPad Prism 8 (GraphPad Software, Inc., CA, USA). We ensured normal distribution using a Shapiro-Wilk normality test. Analysis was done using unpaired Student’s t tests when comparing two variables at a single time point, or one-way ANOVA with sequential post hoc Bonferroni corrections. Results with p values lower than 0.05 were considered as significantly different. p < 0.05 (*), p < 0.01 (**), p < 0.001 (***). Data is shown as mean ± standard error of the mean (SEM). Basic statistical information can be found in figure legend. More detailed information including exact value can be found in supplementary data excel file.

## Supplementary Material

Data S1

Data S2

Supplemental information

## Data Availability

The authors confirm that the data supporting the findings of this study are available within the article [and/or] its supplementary material. RNA-seq data have been deposited at GEO and are publicly available, and accession numbers are listed in the key resources table. All original code has been deposited at Zenodo and is publicly available as of the date of publication. DOIs are listed in the key resources table. Any additional information required to reanalyze the data reported in this paper is available from the lead contact upon request.

## Highlights

- SETD1A deficiency leads to increased synchronization in human neuronal networks
- Structurally, SETD1A deficiency results in increased neuronal dendritic complexity
- Functionally, glutamatergic neurons are the main contributors to the network phenotype
- Mechanistically, SETD1A deficiency is associated with increased cAMP signaling

## In brief

Using human iPSC-derived glutamatergic/GABAergic neuronal cultures, Wang et al. show that loss-of-function variants in *SETD1A* lead to morphologically increased dendritic complexity and functionally increased bursting activity. They demonstrate that *SETD1A* haploinsufficiency leads to increased cAMP signaling, which could be rescued pharmacologically.
