# Supplementary material for: Loss-of-function variants in the schizophrenia risk gene SETD1A alter neuronal network activity in human neurons through the cAMP/PKA pathway
Source: Cell Rep. Author manuscript; Available in PMC 2024 Apr 2. (PMC7615788; doi:10.1016/j.celrep.2022.110790)
Supplement: Supplemental information [file EMS194774-supplement-Supplemental_information.pdf]

Cell Reports, Volume *39*

Supplemental information

Loss-of-function variants in the schizophrenia risk gene *SETD1A* alter neuronal network activity in human neurons through the cAMP/PKA pathway

Shan Wang, Jon-Ruben van Rhijn, Ibrahim Akkouh, Naoki Kogo, Nadine Maas, Anna Bleeck, Irene Santisteban Ortiz, Elly Lewerissa, Ka Man Wu, Chantal Schoenmaker, Srdjan Djurovic, Hans van Bokhoven, Tjitske Kleefstra, Nael Nadif Kasri, and Dirk Schubert

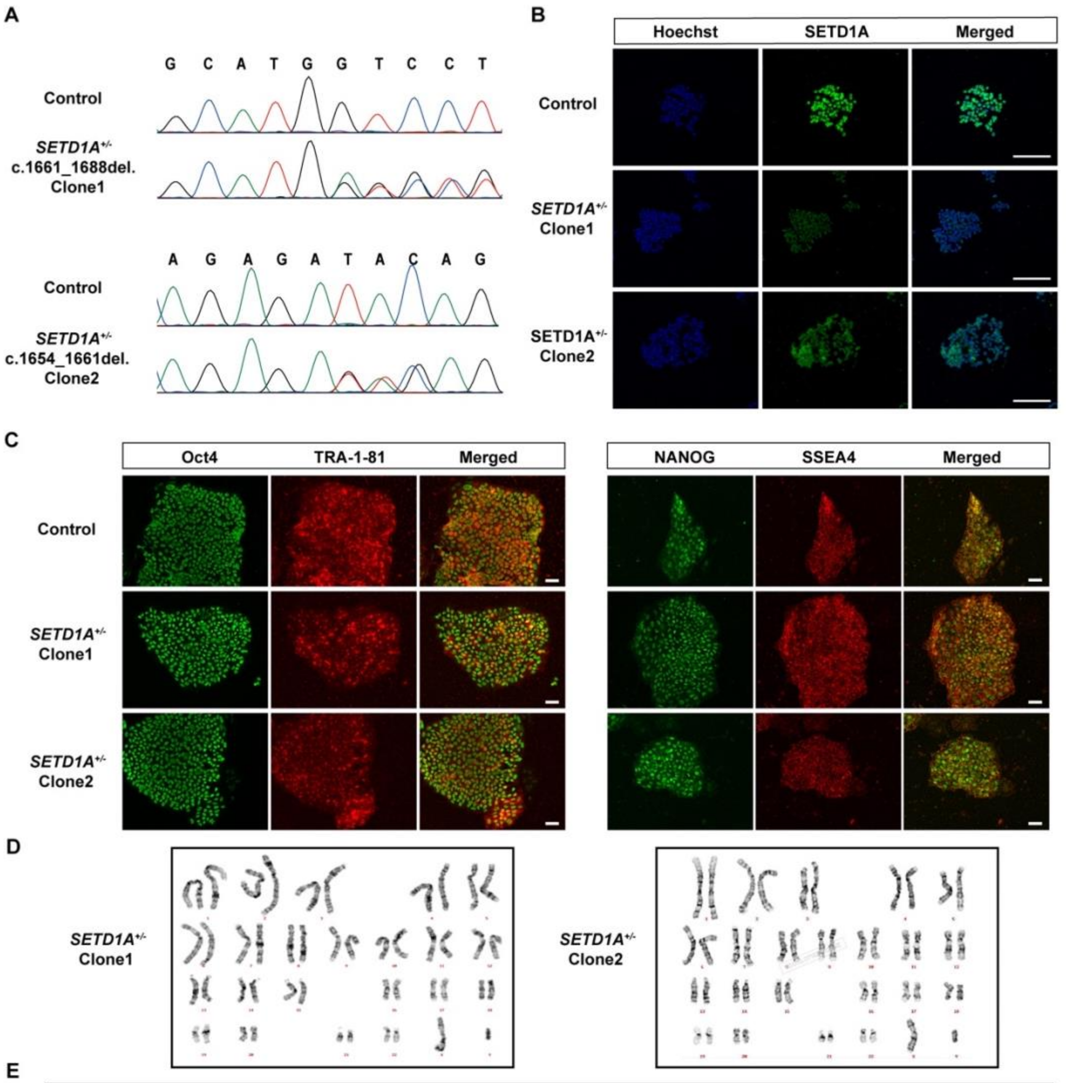

E

Gene	Chr	Position	Strand	Sequence	# mismatch	Score	On target	Primers
LINC00485	16	103212012	-1	CCTGTATCTCTGGCCTCAGGTAC	3	0.86	False	Fw: ATCCCCACATGTGCAGTGAG<br>Rv: TAGTCCTCAAGAGTCTGGGCT
None	1	48188845	-1	CCTGTTTCTCTGGCCCCAAGGTC	2	0.56	False	Fw: CGAGGAGGTTGGGGTGTCAAG<br>Rv: CGCTCCAACATATGCTGTGC
KIAA1671	22	25388422	-1	CCTGTATCTCGGCTCCTAGACAG	3	0.48	False	Fw: CAGGGTCTCAGATGGAGGGA<br>Rv: TCCAGGTGATAACTGCTGGC
BNIP	5	172590193	-1	CCAGTATCCCTGGCCTGAGGGA	4	0.44	False	Fw: GTTCTGACCTCCAGCTGACC<br>Rv: CAGCCAAATGCACCAGAACC
PCLO	7	82581887	1	GTCTCAGTGGCCAGAGATACAGG	4	043	False	Fw: TTCCCTGGGATACGGTGCTA<br>Rv: TCAAGCAGCTCCCACATCAG

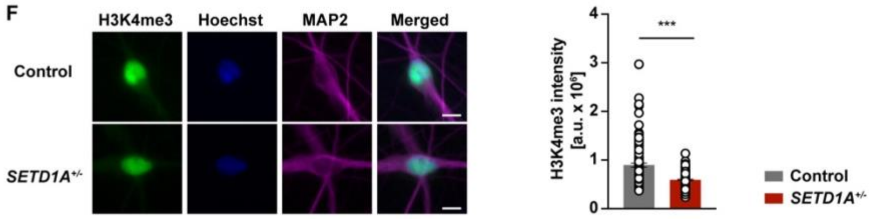

Supplementary figure 1. **Characterization of control and *SETD1A*$^{+/-}$ iPSCs. Related to Figure 1. (a)** Sanger sequence of control and *SETD1A*$^{+/-}$ iPSCs. **(b)** Representative images showing reduced expression of SETD1A in *SETD1A*$^{+/-}$ iPSCs. Scale bar = 100 µm. **(c)** Representative images of control and *SETD1A*$^{+/-}$ iPSCs stained for pluripotent markers. Scale bar = 50 µm. **(d)** *SETD1A*$^{+/-}$ iPSCs maintained a normal karyotype. **(e)** Top five potential off-target sites have been sequenced and no

mutations were detected for both clone 1 and clone 2. **(f)** Representative figures of H3K4me3 (green) and MAP2 (purple) from iNeurons at DIV49 (scale bar = 10 μm) and quantification of H3K4me3 intensity from control (n = 124 cells) and *SETD1A*$^{+/-}$ iNeurons (n = 121 cells from clone 1). ***p < 0.001, unpaired Student's t test was performed between control and *SETD1A*$^{+/-}$ cultures.

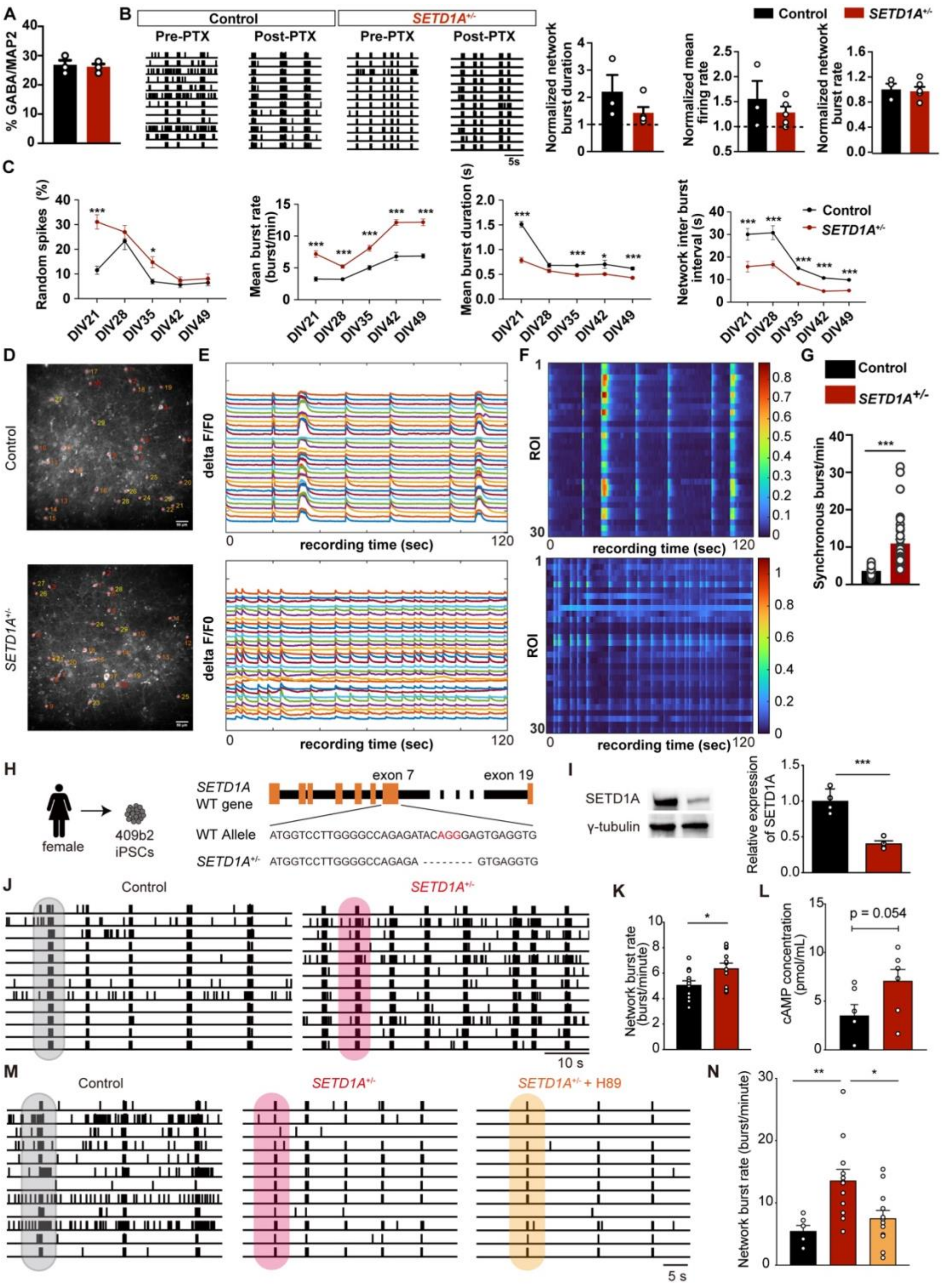

Supplementary figure 2. ***SETD1A***$^{+/-}$ **neurons exhibit dysregulated neuronal network activity, Related to Figure 1. (a)** Quantification of MAP2 and GABA co-localized neurons in networks derived

from control and *SETD1A*$^{+/-}$ iPSCs at DIV49. **(b)** Representative raster plots of 20 sec showing the effect of acute treatment of 100 μM picrotoxin on control and *SETD1A*$^{+/-}$ E/I networks at DIV49; Quantification of network burst duration, mean firing rate and network burst rate normalized to their respective baseline recording. n = 3 individual wells for control. n = 5 individual wells for *SETD1A*$^{+/-}$ from clone 1. **(c)** Quantification of network parameters using MEA recording as indicated. **(d)** Representative ROIs in the field of view showing calcium fluctuations in control (upper panel) and *SETD1A*$^{+/-}$ (lower panel) neurons at DIV70. Scale bar = 50 μm. Representative videos can be seen from Supplementary Video 1 for control and Supplementary Video 2 for *SETD1A*$^{+/-}$. **(e-f)** Changes in calcium signal from 30 neurons showed by temporal calcium traces (e) and heatmap (f) in a 2 min recording from control (upper panel) and *SETD1A*$^{+/-}$ (lower panel) neurons. **(g)** Quantification of synchronous burst rate. n = 23 for control. n = 34 for *SETD1A*$^{+/-}$ from clone 1. *p < 0.05, **p < 0.01, ***p < 0.001, unpaired Student's t test was performed between control and *SETD1A*$^{+/-}$ cultures. *SETD1A*$^{+/-}$ neurons in 409b2 background exhibit dysregulated neuronal network activity and this network phenotype can be rescued by H89 (h-n). **(h)** Generation of *SETD1A* isogenic line using CRISPR/Cas9 in 409b2 iPSC line. Schematic diagram showing position of sgRNA and indels generated in *SETD1A*$^{+/-}$ iPSC line. **(i)** Western blot showing reduced SETD1A expression in *SETD1A*$^{+/-}$ iPSC line. Control: n = 4, *SETD1A*$^{+/-}$ n = 4. **(j)** Representative raster plots of electrophysiological activity exhibited by control and *SETD1A*$^{+/-}$ neuronal networks at DIV49. Gray and pink shadows show the examples of network burst event. **(k)** Quantification of network burst rate. Sample size: control n = 12 MEA wells. *SETD1A*$^{+/-}$ n = 12 MEA wells. **(l)** cAMP concentrations for control (n = 6) and *SETD1A*$^{+/-}$ cultures (n = 7). **(m-n)** Representative raster plot for 30 sec recorded by MEA (m) and quantification of network burst rate (n) showing the effect of PKA inhibitor H89 (2 μM) *SETD1A*$^{+/-}$ cultures (sample size: control n = 6 wells, *SETD1A*$^{+/-}$ + H89 n = 12 wells, *SETD1A*$^{+/-}$ n = 12 wells). Data represent means ± SEM. *P <0.05, **P < 0.01, ***P < 0.001, one-way ANOVA test and post-hoc Bonferroni correction.

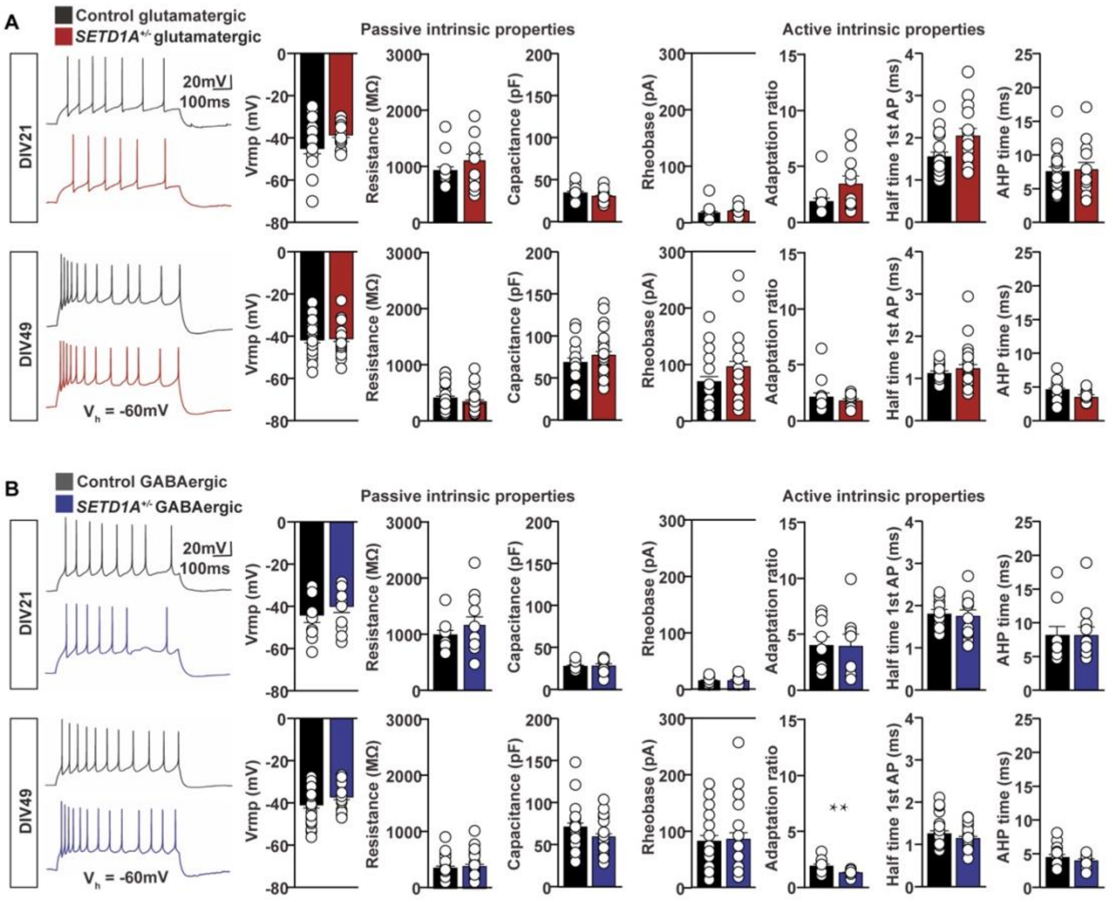

Supplementary figure 3. Basic passive and active intrinsic electrophysiological properties of control and *SETD1A*$^{+/-}$ neurons in E/I networks during development. Related to Figure 1. (a) Glutamatergic neurons: Representative whole-cell patch-clamp recordings of action potential firing pattern and quantitative analyses of basic intrinsic properties of control and *SETD1A*$^{+/-}$ at DIV 21 and DIV49. DIV 21: Control (passive properties n = 18, active properties n = 15), *SETD1A*$^{+/-}$ (passive properties n = 15, active properties n = 11 from clone 1). DIV 49: Control (passive properties n = 28, active properties n = 15), *SETD1A*$^{+/-}$ (passive properties n = 27 (clone 1 = 13, clone 2 = 14), active properties n = 12 from clone 1). **(b)** GABAergic neurons: Representative whole-cell patch-clamp recordings of action potential firing pattern and quantitative analyses of basic intrinsic properties of control and *SETD1A*$^{+/-}$ glutamatergic neurons at DIV 21 and DIV49. DIV 21: Control (passive properties n = 11, active properties n = 9), *SETD1A*$^{+/-}$ (passive properties n = 11, active properties n = 8). DIV 49: Control (passive properties n = 27, active properties n = 14), *SETD1A*$^{+/-}$ (passive properties n = 27 (clone 1 = 13, clone 2 = 14), active properties n = 11 from clone 1). Groups were compared using Students' T-test with Bonferroni correction for multiple testing. Except for the resting membrane potential (Vrmp), parameters were determined at a holding potential (V_h) of -60 mV. Action potential (AP), after-

hyperpolarization (AHP).

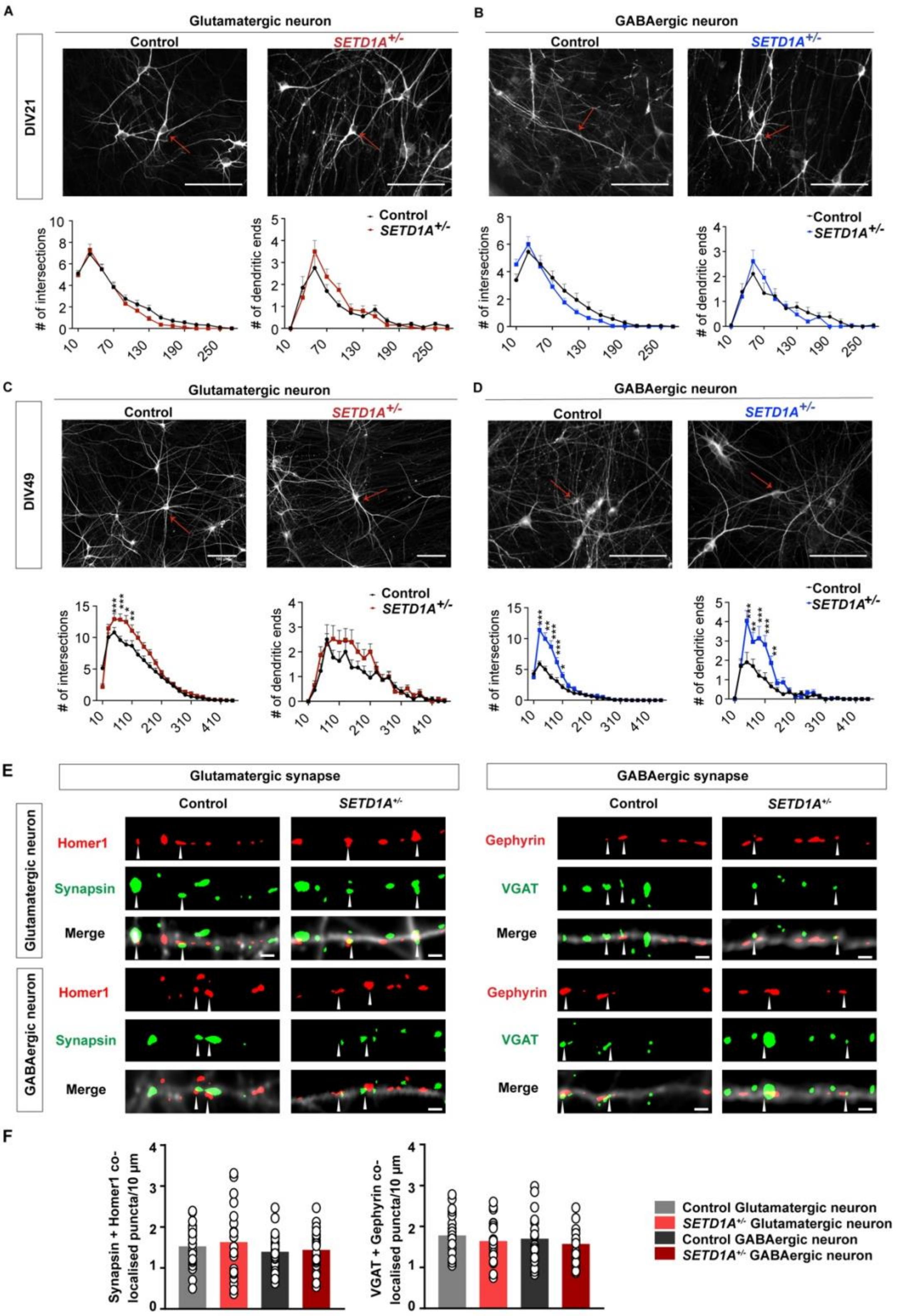

Supplementary figure 4: **Reconstruction and immunocytochemistry results related to Figure 2. (a-d)** Quantitative morphometrical analyse parameters related to reconstructions in Figure 2. Representative somatodendritic reconstructions of glutamatergic (a,c) or GABAergic (b,d) neurons and Sholl analysis

in control and *SETD1A*$^{+/-}$ neurons at DIV21 and DIV49. Red arrows indicate the neurons selected for reconstructions in the Figure 2. Scale bar = 100 µm. **(e)** Representative images of immunocytochemistry stained for glutamatergic synapse (Synapsin as a presynaptic marker and Homer1 as a postsynaptic marker) and GABAergic synapse (VGAT as a pre-synaptic marker and Gephyrin as a post synaptic marker) at DIV49 on each cell type. White arrows indicate the co-localized Synapsin/Homer1 or VGAT/Gephyrin puncta. Scale bar = 2 µm **(f)** Quantification of the density of co-localized Synapsin/Homer1 and VGAT/Gephyrin puncta on glutamatergic neurons (Synapsin/Homer1: n = 22 for control, n = 23 for *SETD1A*$^{+/-}$ from clone 1, VGAT/Gephyrin: n = 22 for control, n = 22 for *SETD1A*$^{+/-}$ from clone 1) and GABAergic neurons (Synapsin/Homer1: n = 20 for control, n = 20 for *SETD1A*$^{+/-}$ from clone 1, VGAT/Gephyrin: n = 20 for control, n = 20 for *SETD1A*$^{+/-}$ from clone 1).

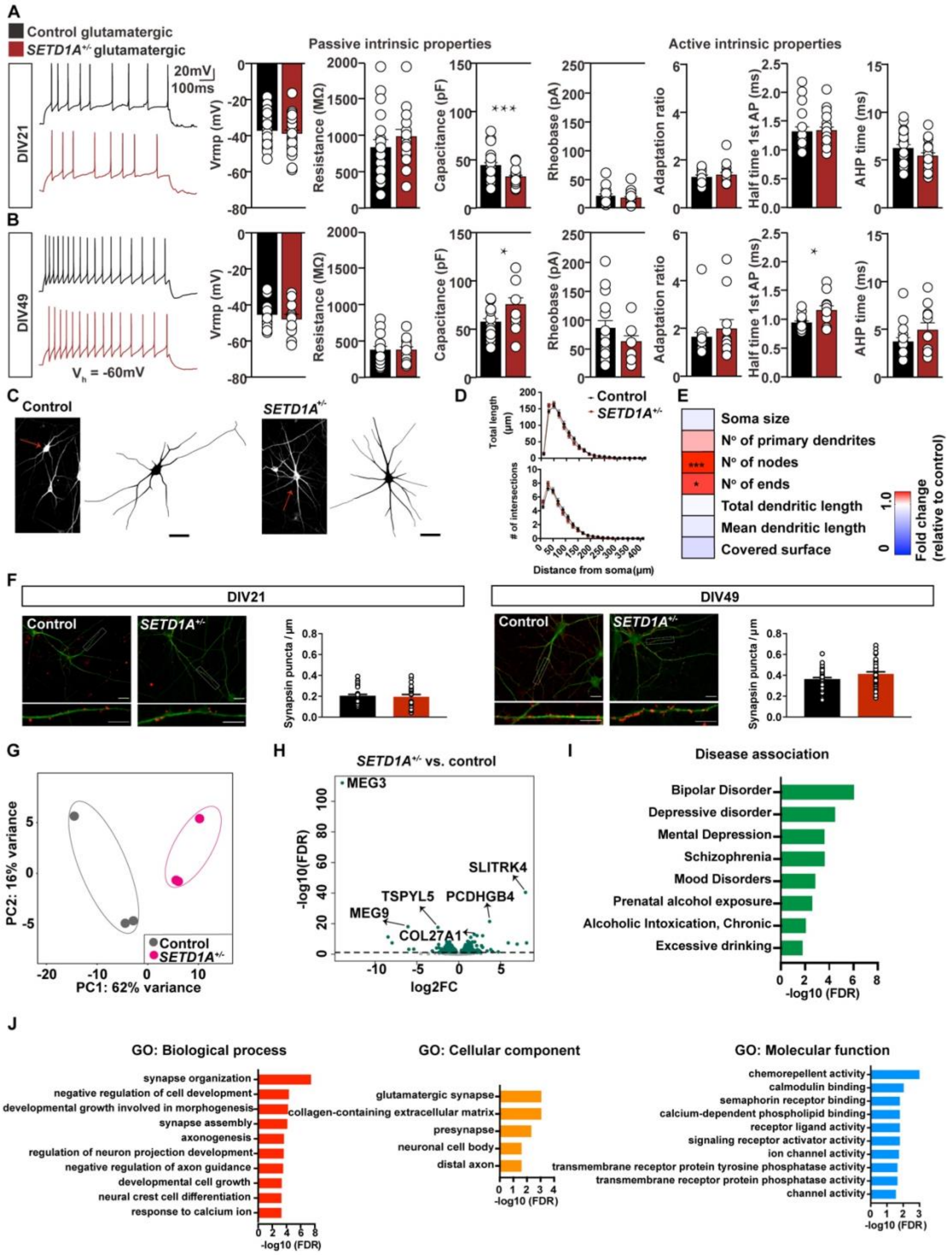

Supplementary figure 5. Electrophysiological, morphological and transcriptomic effect of *SETD1A* haploinsufficiency in glutamatergic neurons. Related to Figure 4. (a) Intrinsic properties of Glutamatergic neurons in glutamatergic cultures at DIV 21. Control/*SETD1A*$^{+/-}$ n = 33/32 (passive properties) and 18/20 (active properties). **(b)** Intrinsic properties of glutamatergic neurons in glutamatergic cultures at DIV 49. Control/*SETD1A*$^{+/-}$ n = 17/12 (passive properties) and 16/11 (active properties). Groups were compared using Students' T-test with Bonferroni correction for multiple testing.

Except for the resting membrane potential (V_{rmp}), parameters were determined at a holding potential (V_h) of -60 mV. Action potential (AP), after-hyperpolarization (AHP). **(c-d)** Representative somatodendritic reconstructions of glutamatergic neurons and Sholl analysis in control and *SETD1A*$^{+/-}$ networks at DIV21. (sample size: n = 45 for control glutamatergic neurons. n = 49 for *SETD1A*$^{+/-}$ glutamatergic neurons) Scale bar = 40 μm. Data represent means ± SEM. *p < 0.05, **p < 0.01, ***p < 0.001, two-way ANOVA with post hoc Bonferroni correction. **(e)** Heatmap showing fold change of all the parameters compared to control in reconstruction for glutamatergic neurons at DIV21. *p < 0.05, **p < 0.01, ***p < 0.001, unpaired Student's t test. **(f)** Representative images of control and *SETD1A*$^{+/-}$ glutamatergic neurons stained for MAP2 (green) and Synapsin (red) at DIV21 and DIV49 (Scale bar = 20 μm) and quantification of Synapsin puncta per μm. (sample size: n = 41 for control glutamatergic neurons. n = 35 for *SETD1A*$^{+/-}$ glutamatergic neurons at DIV21 from clone 1; n = 37 for control glutamatergic neurons. n = 36 for *SETD1A*$^{+/-}$ glutamatergic neurons at DIV49 from clone 1). **(g)** PCA showing tight clustering of 3 replicates for each genotype from RNA-seq analysis. **(h)** Volcano plots of -log10 (FDR) versus the log2 (fold change) of transcript levels for all genes. Relative to control, significantly up or down-regulated genes are shown in green. Top 3 upregulated and downregulated genes are labeled. **(i)** Disease terms of DisGeNET database associated with differentially expressed genes (DEGs). **(j)** Gene Ontology (GO) term analysis of DEGs.

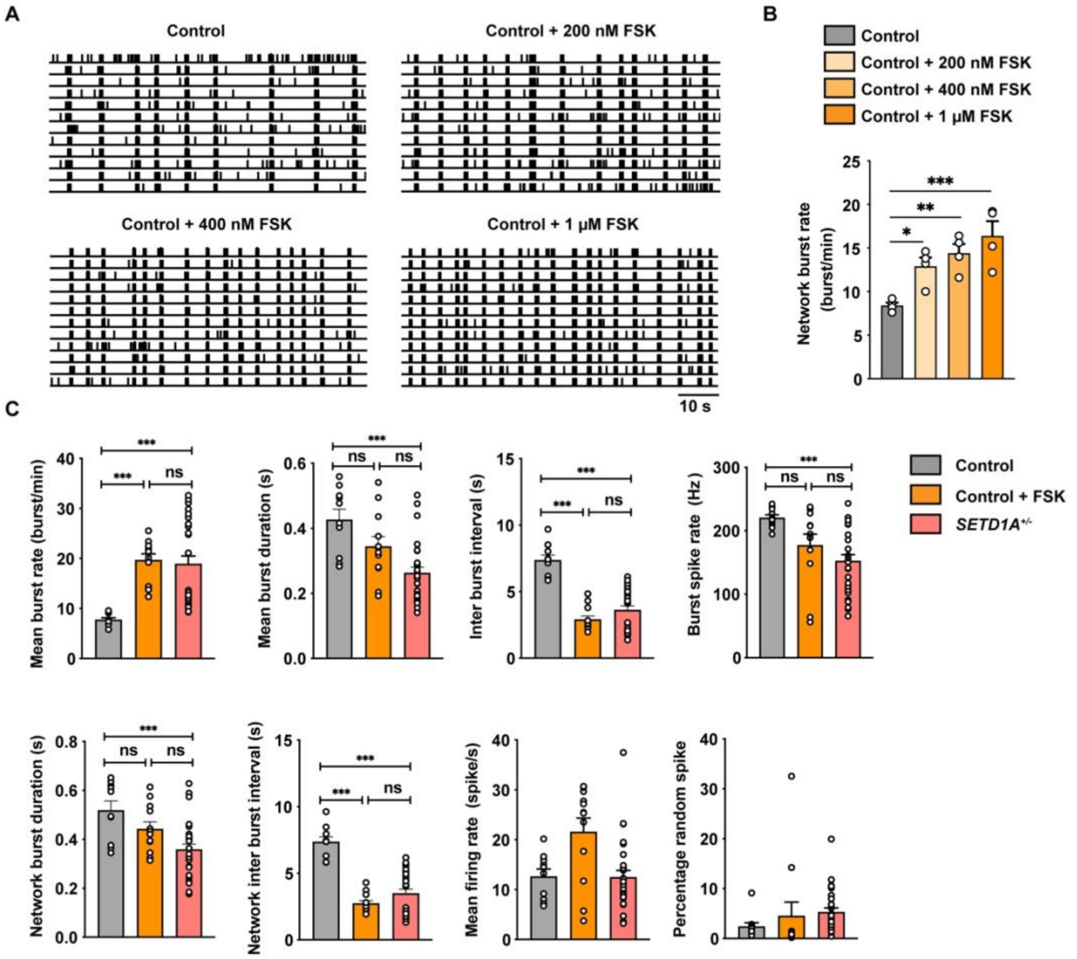

Supplementary figure 6. Control networks treated with Forskolin mimic the phenotype of *SETD1A*$^{+/-}$ networks. Related to Figure 5. (a-b) Representative raster plot for 1 min and quantification of network burst rate showing the dose-dependent effect of AC agonist forskolin on control networks (sample size: control n= 4 wells, control + forskolin n = 4 wells) **(c)** Control networks were treated with 1 µM forskolin for 1 hour. Quantification of network parameters recorded by MEA as indicated. (sample size: control n =10 wells, control + forskolin n = 12 wells, *SETD1A*$^{+/-}$ n = 28 wells). Data represent means ± SEM. *P <0.05, **P < 0.01, ***P < 0.001, one-way ANOVA test with post hoc Bonferroni correction.

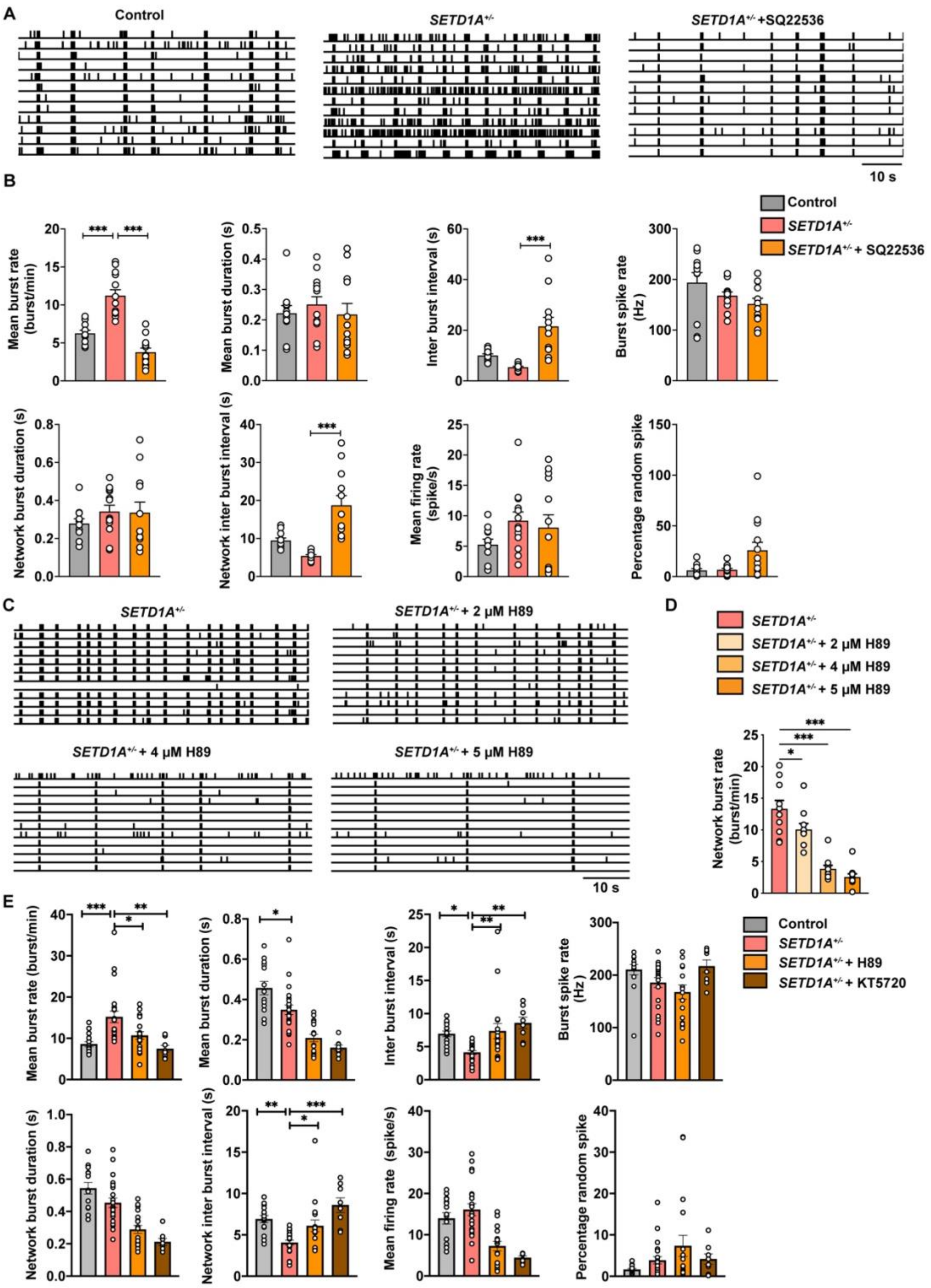

Supplementary figure 7. The phenotype of *SETD1A*$^{+/-}$ networks can be rescued by SQ22536 and H89 or KT5720. Related to Figure 5. (a-b) Representative raster plot for 1 min and quantification of network burst rate showing the effect of adenylate cyclase inhibitor SQ22536 (100 μM) on *SETD1A*$^{+/-}$ networks (sample size: control n = 11 wells, *SETD1A*$^{+/-}$ + SQ22536 n = 13 wells, *SETD1A*$^{+/-}$ n = 13 wells). **(c-d)** Representative raster plot for 1 min and quantification of network burst rate showing the dose-dependent effect of PKA inhibitor H89 on *SETD1A*$^{+/-}$ network. (sample size: *SETD1A*$^{+/-}$ n = 10

wells, *SETD1A*$^{+/-}$ + H89 n = 10 wells) **(e)** *SETD1A*$^{+/-}$ networks were treated with 2 µM H89 or 1 µM KT5720 for 1 hour. Quantification of network parameters recorded by MEAs as indicated. (sample size: control n = 15 wells, *SETD1A*$^{+/-}$ + H89 n = 18 wells, *SETD1A*$^{+/-}$ + KT5720 n = 8 wells, *SETD1A*$^{+/-}$ n = 22 wells). Data represent means ± SEM. *P < 0.05, **P < 0.01, ***P < 0.001, one-way ANOVA test with post hoc Bonferroni correction.

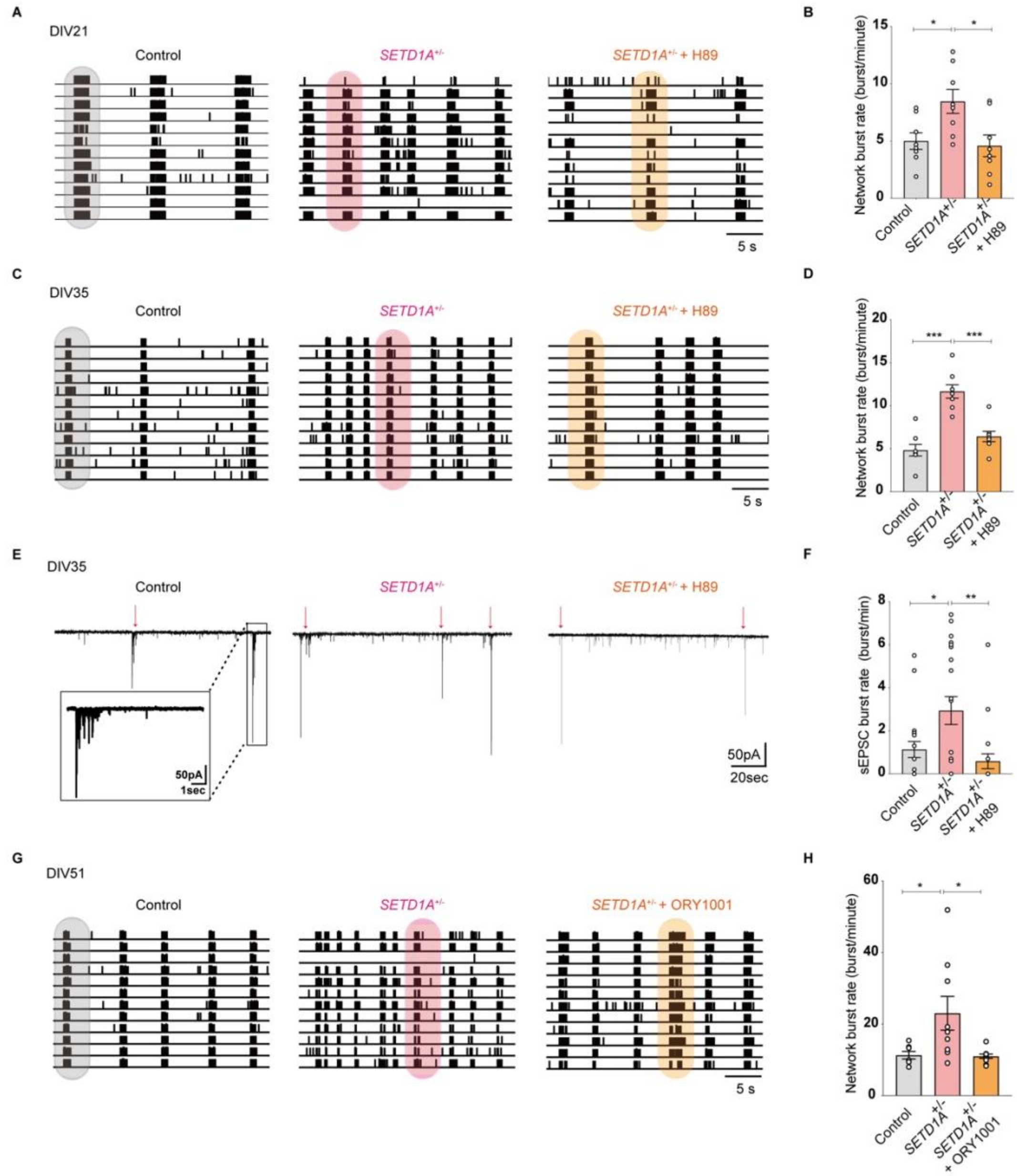

Supplementary figure 8. The phenotype of *SETD1A*$^{+/-}$ networks can be rescued by H89 and ORY1001. Related to Figure 5(l-m). (a-b) Representative raster plot and quantification of network burst rate showing the effect of PKA inhibitor H89 (2 µM) on *SETD1A*$^{+/-}$ networks at DIV 21 (sample size: control n = 8 wells, *SETD1A*$^{+/-}$ + H89 n = 8 wells, *SETD1A*$^{+/-}$ n = 8 wells from clone 1). **(c-d)** Representative raster plot and quantification of network burst rate showing the effect of PKA inhibitor

H89 (2 µM) on *SETD1A*$^{+/-}$ networks at DIV 35 (sample size: control n = 8 wells, *SETD1A*$^{+/-}$ + H89 n = 8 wells, *SETD1A*$^{+/-}$ n = 8 wells from clone 1). **(e-f)** Representative traces and quantification of spontaneous excitatory postsynaptic current burst showing the effect of PKA inhibitor H89 (2 µM) on *SETD1A*$^{+/-}$ cultures at DIV 35 (red arrows indicate burst events). (sample size: control n = 19 cells, *SETD1A*$^{+/-}$ + H89 n = 19 cells, *SETD1A*$^{+/-}$ n = 20 cells from clone 1). **(g-h)** Representative raster plot and quantification of network burst rate showing the effect of LSD1 inhibitor ORY1001 (1 µM) on *SETD1A*$^{+/-}$ networks at DIV 51 (ORY1001 treatment started from DIV10) (sample size: control n = 7 wells, *SETD1A*$^{+/-}$ + ORY1001 n = 10 wells, *SETD1A*$^{+/-}$ n = 9 wells from clone 1). Data represent means ± SEM. *P < 0.05, **P < 0.01, ***P < 0.001, one-way ANOVA test with post hoc Bonferroni correction.

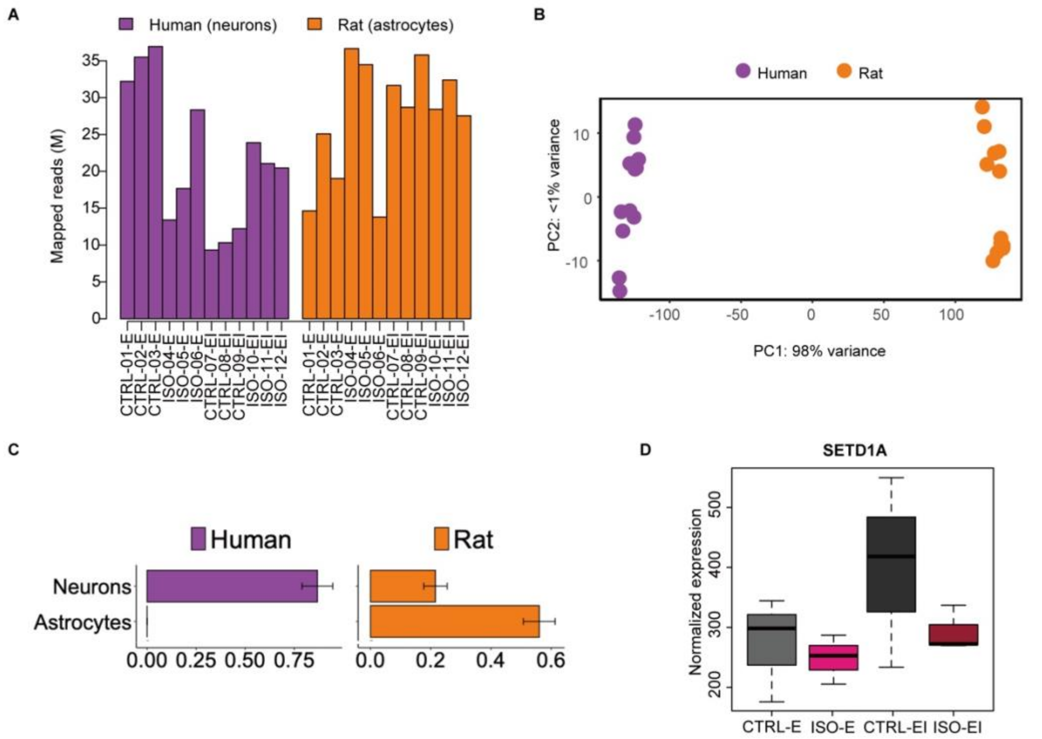

Supplementary figure 9. Separation of mixed-species RNA-seq reads according to species of origin. Related to RNA-seq (Figure 3 and S5 and STAR Methods). (a) Mapped reads (in millions) per sample per species after bioinformatically separating human neuronal reads from rat astrocytic reads. Average sequencing depth per sample (sum of rat and human reads) was 49.1 million reads. **(b)** PCA plot of samples showing complete separation of clusters according to species of origin. Top 1000 genes with largest variance across samples were used as input. **(c)** Deconvolution of cell type proportions showing no contamination of astrocytic reads in human samples after species separation. **(d)** Expression of *SETD1A* from RNA-seq for control and *SETD1A*$^{+/-}$ (isogenic) cultures.

Supplementary table 1: Cell type specific electrophysiological properties of neurons in E/I cultures during development. Related to Figure 1. Statistical testing compared genotypes per cell class and DIV.* = based on KS-test on cumulative data.

**E/I co-culture**	**Glutamatergic neurons**			**GABAergic neurons**		
**DIV 21**	Control	*SETD1A*$^{+/-}$	p-value	Control	*SETD1A*$^{+/-}$	p-value
**Passive intrinsic properties**	n = 18	n = 15		n = 27	n = 27	
Resting membrane potential (mV)	-45.1 ± 3.1	-38.5 ± 1.3		-46.6 ± 3.6	-42.1 ± 3.1	
Membrane resistance (MΩ)	976 ± 68	1157 ± 123		1044 ± 84	1223.0 ± 161	
Membrane capacitance (pF)	36.7 ± 1.8	32.2 ± 2.0		28.9 ± 1.5	29.3 ± 2.7	
**Active intrinsic properties**	(n = 15)	(n = 11)	p-value	(n = 14)	(n = 11)	p-value
AP threshold (mV)	-27.9 ± 1.1	-25.3 ± 1.4		-26.6 ± 1.3	-27.0 ± 1.4	
Rheobase (pA)	18.1 ± 2.7	21.3 ± 2.3		17.5 ± 1.9	17.7 ± 2.3	
1st AP amplitude	98.4 ± 2.2	98.7 ± 3.4		99.2 ± 2.6	99.6 ± 4.8	
1st AP Half time (ms)	1.6 ± 0.1	2.0 ± 0.2		1.9 ± 0.1	1.8 ± 0.2	
Adaptation rate 8-9rd/2-3rd AP	2.0 ± 0.3	3.6 ± 0.7		4.2 ± 0.8	4.1 ± 1.1	
1st AP AHP time (ms)	8.0 ± 0.8	8.3 ± 1.2		8.6 ± 1.4	8.6 ± 1.3	
1st-2nd AP AHP amplitude (mV)	2.8 ± 0.5	0.5 ± 1.4		0.8 ± 1.1	0.6 ± 0.9	
**Synaptic inputs/ postsynaptic events**	(n = 23)	(n = 22)	p-value	(n = 21)	(n = 22)	p-value
sEPSC amplitude (pA)	31.0 ± 2.0	30.3 ± 2.6		28.9 ± 3.9	25.6 ± 1.6	
sEPSC frequency (Hz)	1.8 ± 0.5	1.9 ± 0.4		1.6 ± 0.3	1.8 ± 0.6	
sEPSC synchronous inputs (n/min)	0.5 ± 0.3	0.7 ± 0.2		1.9 ± 0.4	1.6 ± 0.4	

DIV 49

Passive intrinsic properties	(n = 28)	(n = 27)	p-value	(n = 27)	(n = 27)	p-value
Resting membrane potential (mV)	-45.3 ± 1.6	-44.6 ± 1.6		-44.4 ± 1.6	-40.3 ± 1.4	
Membrane resistance (MΩ)	435.0 ± 44.4	361.0 ± 42.9		386.3 ± 38.5	415.3 ± 46.2	
Membrane capacitance (pF)	73.5 ± 4.6	82.9 ± 6.0		76.6 ± 5.2	64.0 ± 4.1	
**Active intrinsic properties**	(n = 15)	(n = 12)	p-value	(n = 14)	(n = 11)	p-value
AP threshold (mV)	-32.0 ± 1.9	-27.7 ± 1.3		-28.8 ± 1.7	-27.0 ± 1.0	
Rheobase (pA)	75.0 ± 9.8	103.8 ± 13.4		89.0 ± 11.0	92.9 ± 12.9	
1st AP amplitude	104.5 ± 2.5	99.4 ± 3.1		104.6 ± 2.7	106.7 ± 2.5	
1st AP Half time (ms)	1.2 ± 0.1	1.3 ± 0.1		1.3 ± 0.1	1.2 ± 0.1	
Adaptation rate 8-9rd/2-3rd AP	2.3 ± 0.4	1.9 ± 0.2		2.0 ± 0.2	1.4 ± 0.1	0.005
1st AP AHP time (ms)	4.9 ± 0.4	3.7 ± 0.3		4.7 ± 0.5	4.1 ± 0.3	
1st-2nd AP AHP amplitude (mV)	1.8 ± 0.3	2.2 ± 0.5		1.7 ± 0.7	1.7 ± 0.7	
**Synaptic inputs/ postsynaptic events**	(n = 15)	(n = 13)	p-value	(n = 18)	(n = 16)	p-value
sEPSC amplitude (pA)	24.8 ± 2.4	22.4 ± 1.0		22.3 ± 1.9	24.0 ± 2.5	
sEPSC frequency (Hz)	2.5 ± 0.7	2.2 ± 0.6		1.6 ± 0.3	1.6 ± 0.4	
sEPSC synchronous inputs (n/min)	1.1 ± 0.2	2.0 ± 0.4	0.031	0.8 ± 0.1	1.6 ± 0.3	0.009
	(n = 17)	(n = 16)	p-value	(n = 16)	(n = 13)	p-value
mEPSC amplitude (pA)	17.3 ± 1.0	20.2 ± 1.5	<0.001*	16.6 ± 1.2	19.5 ± 2.1	<0.001*

mEPSC frequency (Hz)	1.4 ± 0.3	2.0 ± 0E.5	<0.001*	1.1 ± 0.3	2.8 ± 0.6	<0.001*

Supplementary table 2: Transcriptomic analyses based on DEGs. Related to Figure 3.

Overrepresented DEGs in "Schizophrenia" from disease enrichment analysis using DisGeNET database	
Schizophrenia	*ABCB1,ADNP,ALDH1A2,APOL2,ASTN2,BACE1,BCL9,CACNG8,CCDC86,CCND2,CDKN1C,CHGA,CHGB,CNIH3,CPLX1,CRH,DCC,DKK3,DLG2,DNMT3B,DRD2,DTNBP1,FABP5,FADS2,FASTKD5,GDNF,GFRA1,GRIK3,GRIN2A,GRM3,GRM4,GSK3A,HDAC2,HLAC,HTR3B,HTR7,KCNB1,KLF12,KMT2A,LPAR1,MAGI1,MAOB,MBP,MET,MYO16,NR3C1,NRXN3,PANK2,PBRM1,PCDH17,PHOX2B,PLCB1,PLXNA2,PNPO,PTPN21,PTPRA,S100B,SLC12A5,SLC25A27,SLC6A2,SLIT3,SNCB,ST3GAL1,SULT4A1,TNFRSF1A,TPI1,UCP2,ZSCAN31*

Term	Upregulated DEGs involve in synaptic strength
Neurotransmitter transport	*DTNBP1,MAOB,SYT17,SLC5A7,PARK7,GRM4,NAPB,PNKD,SNAP25,VAMP1,SYT2,CRH,DRD2,CADPS,GDNF,CPLX1,TRH,BACE1,VAMP2,PRAF2*
Neurotransmitter secretion	*DTNBP1,SYT17,SLC5A7,GRM4,NAPB,PNKD,SNAP25,VAMP1,SYT2,DRD2,CADPS,CPLX1,BACE1,VAMP2*
Synaptic vesicle cycle	*DTNBP1,SNCB,AMPH,SYNDIG1,SYT17,PACSIN1,NAPB,SNAP25,VAMP1,SYT2,DRD2,CADPS,CPLX1,BACE1,VAMP2*
Synaptic vesicle exocytosis	*DTNBP1,SYT17,NAPB,SNAP25,VAMP1,SYT2,DRD2,CADPS,CPLX1,BACE1,VAMP2*
Vesicle-mediated transport in synapse	*DTNBP1,SNCB,AMPH,SYNDIG1,SYT17,PACSIN1,NAPB,SNAP25,VAMP1,SYT2,DRD2,CADPS,CPLX1,BACE1,VAMP2*

Comparison of DEGs with transcriptomic profile of the published *Setd1a*$^{+/-}$ mice	
Comparison with published datasets	Upregulated genes: *SLITRK4,PDZD2,POSTN,RASGEF1B,ATP6V1F,UCP2,PARVA*

(Mukai et al, 2019)	Down-regulated genes: *ARID4A,FGF11,KMT2A,ZNF462*

Comparison of DEGs with SFARI datasets	
Overlap with SFARI genes	*ADNP,ALDH1A3,ASTN2,ASXL3,ATP1A3,ATP2B2,CACNA2D3,CADPS,CAMK4,CDH8,CDH9,CELF6,CEP290,CHD3,CHRM3,CPEB4,CSNK2A1,DCC,DLG2,DLX6,DPYSL3,DRD2,EPC2,FABP5,FHIT,FOXG1,GLRA2,GPR37,GRIK3,GRIN2A,HLADPB1,IL1RAPL1,INPP1,KCNB1,KCND2,KCTD13,KDM5B,KIRREL3,KMT2A,LAMB1,LRRC4C,MACROD2,MAOB,MBD5,MEGF11,MET,MKX,MRTFB,MYO16,NEGR1,NEO1,NINL,NRXN3,NUAK1,PATJ,PCDHA10,PCDHA4,PCDHA6,PLCB1,PRICKLE2,RALGAPB,RBFOX1,RPS6KA2,SATB1,SH3RF3,SLC12A5,SLC25A27,SLC9A9,SNAP25,STAG1,SYT17,TSHZ3,TTN,UNC5D,VAMP2,ZMYND8,ZNF462,ZNF827*

Supplementary table 3: Cell type specific electrophysiological properties of neurons in glutamatergic cultures during development. Statistical testing compared genotypes per cell class and DIV.* = based on KS-test on cumulative data. Related to Figure 4.

**Glutamatergic cultures**		
**DIV 21**	Control	*SETD1A*$^{+/-}$	
**Passive intrinsic properties**	(n = 33)	(n = 32)	p-value
Resting membrane potential (mV)	-36.4 ± 1.4	-38.0 ± 1.8	
Membrane resistance (MΩ)	827 ± 64	967.0 ± 52	
Membrane capacitance (pF)	44.1 ± 3.0	32.3 ± 1.5	<0.001
**Active intrinsic properties**	(n = 18)	(n = 20)	p-value
AP threshold (mV)	-30.7 ± 1.4	-29.4 ± 1.5	
Rheobase (pA)	20.1 ± 3.0	16.9 ± 2.2	
1st AP amplitude	107.3 ± 1.7	107.8 ± 2.4	
1st AP Half time (ms)	1.4 ± 0.1	1.4 ± 0.1	
Adaptation rate 8-9rd/2-3rd AP	1.4 ± 0.1	1.5 ± 0.1	

1st AP AHP time (ms)	6.4	±	0.4	5.6	±	0.4	
1st-2nd AP AHP amplitude (mV)	0.1	±	0.4	0.4	±	0.5	
**Synaptic inputs/ postsynaptic events**	(n = 27)			(n = 23)			p-value
sEPSC amplitude (pA)	29.3	±	2.6	26.4	±	2.2	0.038
sEPSC frequency (Hz)	3.4	±	0.5	3.6	±	0.8	
sEPSC synchronous inputs (n/min)	1.1	±	0.2	3.2	±	0.5	<0.001

DIV 49

**Passive intrinsic properties**	(n = 17)			(n = 12)			p-value
Resting membrane potential (mV)	-45.0	±	1.9	-47.5	±	2.6	
Membrane resistance (MΩ)	368.8	±	51.4	367.5	±	47.4	
Membrane capacitance (pF)	56.6	±	3.7	74.8	±	7.2	0.020
**Active intrinsic properties**	(n = 16)			(n = 11)			p-value
AP threshold (mV)	-33.3	±	1.2	-29.1	±	0.9	0.020
Rheobase (pA)	84.1	±	13.4	60.9	±	0.1	
1st AP amplitude	103.0	±	1.3	98.5	±	2.5	
1st AP Half time (ms)	1.0	±	0.1	1.2	±	0.1	0.010
Adaptation rate 8-9rd/2-3rd AP	1.7	±	0.2	2.1	±	0.4	
1st AP AHP time (ms)	3.9	±	0.5	5.2	±	0.8	
1st-2nd AP AHP amplitude (mV)	-1.0	±	0.6	0.1	±	0.8	
**Synaptic inputs/ postsynaptic events**	(n = 9)			(n = 12)			p-value
sEPSC amplitude (pA)	30.4	±	2.8	28.1	±	3.0	
sEPSC frequency (Hz)	1.3	±	0.4	2.3	±	0.4	
sEPSC synchronous inputs (n/min)	0.8	±	0.3	5.9	±	1.0	<0.001

Supplementary table 4: Upregulated DEGs involve in second messenger signaling. Related to Figure 5a.

Term	List of genes
G protein-coupled receptor signaling pathway, coupled to cyclic nucleotide second messenger	*ADCY2,PTHLH,CHGA,GSK3A,GLP1R,GHRH,GRM4,RAMP1,SSTR1,HTR7,DRD2,ADCY8,GRIK3,GPR37,ADGRG2,GRM3*
Adenylate cyclase-modulating G protein-coupled receptor signaling pathway	*ADCY2,PTHLH,CHGA,GSK3A,GLP1R,GHRH,GRM4,RAMP1,DRD2,ADCY8,GRIK3,GPR37,ADGRG2,GRM3*
Second-messenger-mediated signaling	*TMEM38A,ADCY2,PTHLH,FKBP1A,SLC9A1,CHGA,NCALD,GSK3A,GLP1R,VEGFA,NR5A2,GHRH,RAMP1,EPHA5,CRH,GSTO1,DRD2,ADCY8,PRNP,NUDT4,ADGRG2,GRIN2A*
